# A Three-Dimensional Analysis of Morphological Evolution and Locomotor Performance of the Carnivoran Forelimb

**DOI:** 10.1371/journal.pone.0085574

**Published:** 2014-01-15

**Authors:** Alberto Martín-Serra, Borja Figueirido, Paul Palmqvist

**Affiliations:** Departamento de Ecología y Geología, Facultad de Ciencias, Universidad de Málaga, Málaga, Spain; University of Lethbridge, Canada

## Abstract

In this study, three-dimensional landmark-based methods of geometric morphometrics are used for estimating the influence of phylogeny, allometry and locomotor performance on forelimb shape in living and extinct carnivorans (Mammalia, Carnivora). The main objective is to investigate morphological convergences towards similar locomotor strategies in the shape of the major forelimb bones. Results indicate that both size and phylogeny have strong effects on the anatomy of all forelimb bones. In contrast, bone shape does not correlate in the living taxa with maximum running speed or daily movement distance, two proxies closely related to locomotor performance. A phylomorphospace approach showed that shape variation in forelimb bones mainly relates to changes in bone robustness. This indicates the presence of biomechanical constraints resulting from opposite demands for energetic efficiency in locomotion –which would require a slender forelimb– and resistance to stress –which would be satisfied by a robust forelimb–. Thus, we interpret that the need of maintaining a trade-off between both functional demands would limit shape variability in forelimb bones. Given that different situations can lead to one or another morphological solution, depending on the specific ecology of taxa, the evolution of forelimb morphology represents a remarkable “one-to-many mapping” case between anatomy and ecology.

## Introduction

Locomotion is crucial for an animal's ecology. Animals move in their home ranges to forage for food resources, to search for mating partners, to avoid stressful environments, to pursue their prey, or to escape from potential predators [1], [2]. Therefore, understanding the way that an animal moves provides a key aspect of its biology and helps to define its role within the ecosystem [3]. For this reason, a major issue for both living and extinct species is to study their locomotor abilities to further understand their sinecological relationships within present and past communities.

Limb indicators of locomotor strategies –i.e., adaptations– in the living taxa may provide highly valuable information to decipher how extinct animals moved. Furthermore, due to the correlation between the mode of locomotion of extant species and the type of habitats in which they inhabit, ecomorphological inferences on the locomotor behavior of extinct taxa provide clues for reconstructing environmental changes in past ecosystems. In this way, during the last decades a number of studies have used a number of shape indicators for estimating the locomotor abilities of extinct mammals [4]–[10] and, more specifically, of mammalian carnivores [11]–[26]. The morphological indicators used were presumed to be the result of selective processes that shaped different anatomical adaptations towards specific modes of locomotion. However, natural selection is not the only factor that shapes morphological traits and it is thus important to quantify the influence of other potential sources of bone variation. An example would be the phylogenetic legacy of a given monophyletic group, a key aspect in phenotypic evolution as it determines the developmental routes and biomaterials available to natural selection [27]–[29]. Similarly, given that the limbs withstand the animal's weight, body mass is one of the most influential factors on limb bone shape [30]–[36]. This is particularly important in the case of the forelimbs, as they withstand the largest amount of body mass in carnivorans [37], [38] and other mammals [39]–[42] relative to the hindlimbs. For these reasons, to explore the influence of phylogeny, body mass and locomotor performance on the shape of the major limb bones is crucial to any study on the evolution of the appendicular skeleton. In addition, this information can be used for deciphering the autecological attributes of extinct taxa and their sinecological roles within past communities.

**Figure 1:**
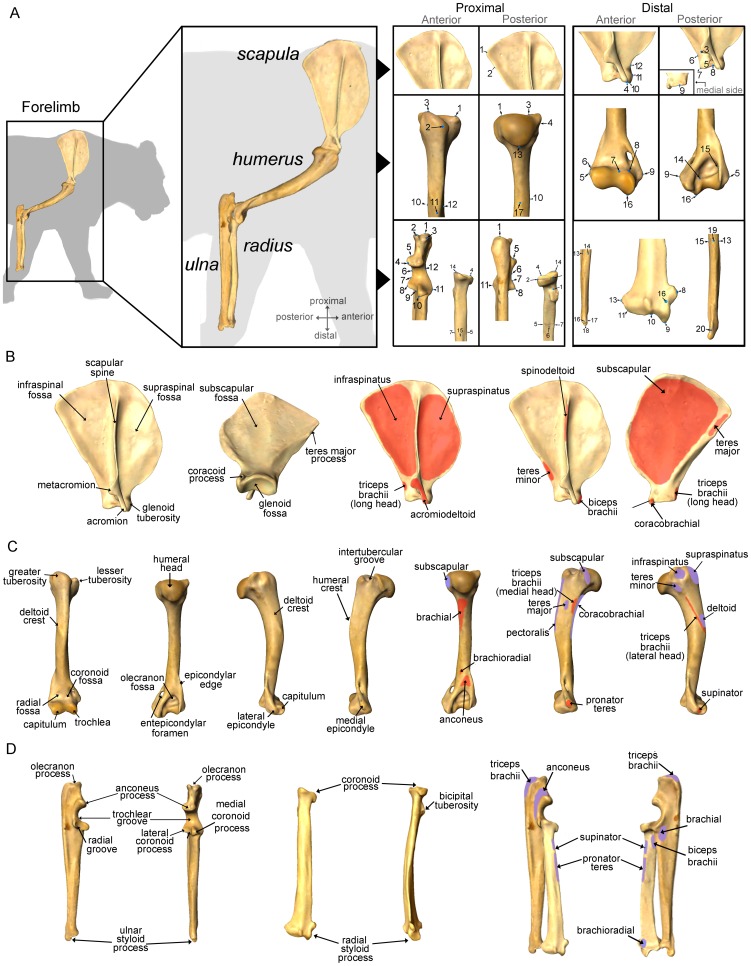
A three-dimensional analysis of forelimb evolution in carnivorans. A, major limb bones analyzed, exemplified on a jaguar (*Panthera onca*), and landmarks used in the morphometric analyses of each forelimb bone. B, morphological key features in the shoulder. C, main morphological structures in the humerus. D, main morphological structures in the radius and ulna of carnivorans. Muscle origins (red) and insertions (purple) for the main muscles involved in locomotion are shown for each forelimb bone (anatomical keys are taken from Barone [50] and Homberger and Walker [51]).

In this article, we: *(i)* quantify the influence of size, phylogeny and locomotor performance in shaping the morphology of forelimb bones in mammalian carnivores; and *(ii)* explore how their morphological variability reflects functional adaptations. The order Carnivora is an excellent choice for this study, as it represents one of the most spectacular cases of repeated and independent evolution of similar morphologies on a limited range of ecologies [12], [13], [16], [26], [43]–[49]. Specifically, we characterized the morphology of the four major bones of the forelimb (i.e., scapula, humerus, radius and ulna; see Figure 1A) using three-dimensional geometric morphometrics methods in order to answer the following questions: *(i)* How important are allometric effects for shaping the morphology of forelimb bones? *(ii)* Is there a phylogenetic structure in all of these bones? *(iii)* Is there a correlation between locomotor performance and the shape of the major forelimb bones? *(iv)* Which are the evolutionary pathways followed by different families? And *(v)* could this information be used in future studies for deciphering how extinct animals moved? We hypothesize that, although the allometry has probably played an important role shaping the limb bones and a strong phylogenetic signal is also expected, the shape of the forelimb bones of carnivorans should also reflect the biomechanical demands posed by their adaptations to different modes of locomotion.

## Materials and Methods

### Data

The data set comprises 138 scapulae, 185 humeri, 230 radii and 186 ulnae (Figure 1A) from 78 species (46 extant and 32 extinct) of the order Carnivora (see File S1: Tables S1, S2 and S3 in File S1). In spite of the fact that this study does not include all living species of the order Carnivora, the species selected cover a high morphological and taxonomic variability within the order, including both living and extinct taxa, in order to avoid biases in subsequent multivariate analyses (e.g., for quantifying the phylogenetic signal). However, it is worth to mention that we have paid special attention to the families with large representatives (i.e., canids, ursids, hyaenids and felids). *Patriofelis* (Mammalia, Creodonta) was incorporated whenever possible as an outgroup for checking how the carnivoran “bauplan” departs from another highly carnivorous mammalian orders such as Creodonta [52]. Data from adult specimens –as indicated by complete distal epiphyseal fusion– were the only collected to avoid potential effects of ontogentic variation. All the specimens analyzed are housed in the following institutions: American Museum of Natural History (AMNH, New York), Natural History Museum (NHM, London), Naturhistorisches Museum (NMB, Basel), Museo Nacional de Ciencias Naturales (MNCN, Madrid), Museo di Storia Naturale (MSN, Firenze), Staten Naturhistoriske Museum (SNM, Copenhagen), Museo de Ciencias Naturales de Valencia (MCNV, Valencia). All of them were analyzed by AMS on loan at their housing institutions with the adequate permissions and under the supervision of the people responsible of those specimens.

### Geometric morphometrics

A set of three-dimensional (3D) homologous landmarks (LK) were digitized by one of us (AM-S) directly to the specimens using a Microscribe G2X. LKs were chosen following different anatomical criteria (File S1: Table S4) for capturing as many morphological aspects as possible in all the bones as well as measuring the most important lever arms for muscle attachments (see Figure 1B–D and Figure S1). It is worth noting that special attention was paid to criteria of homology in the LKs digitized. For this reason, geometric criteria –e.g., tips of epicondyles, processes or tuberosities– prevailed in the selection of LKs over others such as muscle attachment areas, which do not reflect in some cases either biological or functional homology, particularly in a wide taxonomic sample. 3D coordinates (*x*, *y*, *z*) of all LKs were recovered into Excel using the software *Immersion Inc*. In addition, the LKs were digitized several times on the forelimb bones of a cat and a fox for testing data repeatability.

The surface of each forelimb bone of a specimen of *Panthera onca* (Carnivora, Felidae) housed at the AMNH was scanned using a 3D-mobile surface scanner (Nextengine HD) and software ScanStudio Pro. Subsequently, 3D-surface models were imported into software *Landmark* [53] from the Institute of Data Analysis and Visualization (IDAV 2002–2006) and the selected LKs were located on them. Afterwards, LKs' coordinates obtained from statistical analyses with software MorphoJ [54] were also imported into software *Landmark*. This allowed the same LKs directly digitized onto the specimens sampled with Microscribe G2X to be also digitized onto the 3D-surface models of the *P. onca* scanned. Once the correspondence of LKs were established, 3D-surface models were transformed using thin-plate splines by morphing into the coordinates of the morphological extremes obtained in each multivariate axis (see below), which allowed to obtain 3D-surface models of these morphological shapes following the same procedures as in Wiley et al. [53], Drake and Klingenberg [55], Schoenebeck et al. [56] and Singleton [57].It is worth noting that although these models do not improve the results obtained, they are useful for visualizing the morphological interpretations derived from morphometric analyses of complex morphological structures. However, it should be noted that the zones between landmarks should be interpreted with caution, as the models for the different carnivoran taxa analyzed are based on the transformation of a single original shape. For a detailed description of the advantages and disadvantages of warping 3D-surface models, see Klingenberg [58].

Given that 3D models have a low operational value in the printed version of the article, an interactive 3D-PDF was computed available at files S3 and S4.

A Procrustes fit [59], [60] was performed separately from the raw coordinates of the LKs digitized on each bone using software MorphoJ [54]. This procedure removes the effects of rotation, translation and scaling [61]. Once the specimens were aligned, Procrustes coordinates and Centroid size (Cs) were both averaged by species in order to avoid the effects of static allometry within the sample. Those fossil specimens that were not identified at the species level or those extinct species without a resolved within-genus phylogenetic relationship (e.g., *Barbourofelis* sp., or *Smilodon* sp.) were averaged by genus to avoid polytomies.

### Assessing the phylogenetic signal in limb bone shape

**Figure 2:**
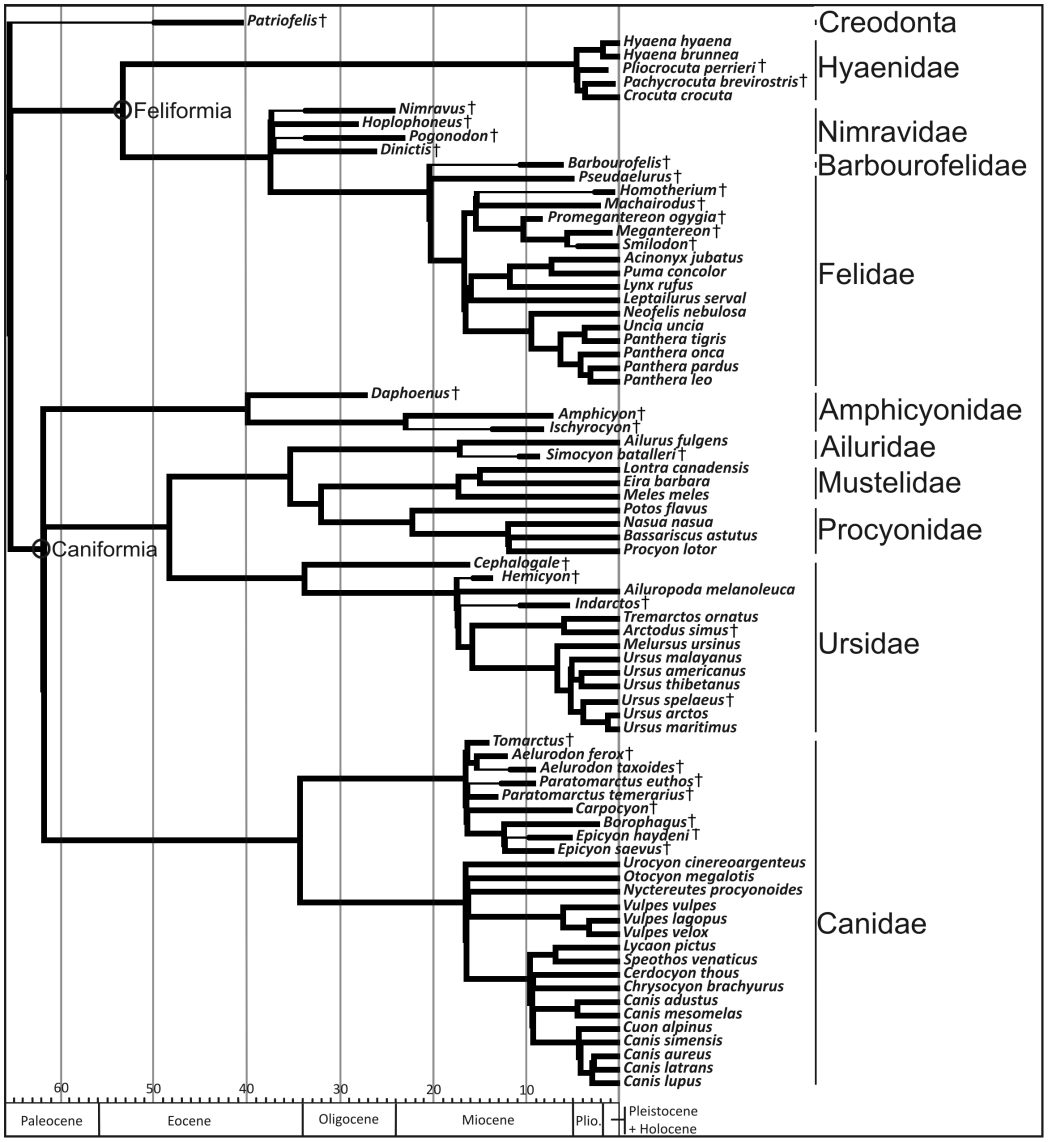
Phylogenetic tree topology for the order Carnivora (plus the creodont *Patriofelis*) used in this study. Thick lines for extinct species indicate stratigraphic range. Detailed references for the tree topology and branch lengths are given in file S1: table S5.

A phylogenetic tree topology based on previously published phylogenies [52], [62]–[75] (see File S1: Table S5 and File S2 for detailed information) was assembled (Figure 2) using Mesquite [76] for assessing the presence of phylogenetic structure in forelimb bones and testing for phylogenetic patterning in multivariate analyses (see below). Branch lengths were incorporated in the composite phylogeny in million years before present for improving the accuracy of the reconstructed ancestral states [69], [77]. Branches were scaled in the living species and groups according to estimates of node dates from the supertree obtained for all members of Carnivora by Nyakatura and Bininda-Emonds [75], using information from Koepfli et al. [70] for procyonids. Fossil occurrence dates were compiled for extinct taxa from various sources based on species locality and age information [63]–[65], [71], including the online databases Paleobiology Database [http://paleodb.org/cgi-bin/bridge.pl?a=home] and the NOW database [http://www.helsinki.fi/science/now/index.html] (see File S1: Table S5 for detailed information). Hence, branch lengths for extinct taxa were estimated from their first and last appearance data. Where estimates of divergence age based on molecular data and on stratigraphic ranges of extinct taxa differed, the oldest dates were chosen.In those cases in which several nodes overlapped at the same date, an arbitrary difference of 0.1 My was introduced between consecutive nodes.

A multivariate regression analysis [82] of the PIC of the Pco of each bone against the SD of the standardized contrast (i.e., the square root of the corrected branch lengths) was performed using MorphoJ [54], following Díaz-Uriarte and Garland [83]. SD values were obtained from the PDAP module for Mesquite [76], [84]. The significance of these multivariate regressions was obtained by a permutation test against the null hypothesis of complete independence between the SD and the PIC of the Pco. This test reshuffles randomly the data (10,000 times) and recalculates the regression. Therefore, the number of random regressions with a correlation between the variables equal or higher than the original one indicates the level of significance. This test was performed in order to explore the adequacy of: (i) the model used for tree topology; (ii) the branch lengths used; and (iii) the model of Brownian motion for our tip data [83].

In order to quantify the presence of phylogenetic signal in both the shape and size of limb bones, a permutation test developed for univariate traits by Laurin [85], extended for multivariate analysis by Klingenberg & Gidaszewski [86] and applied to size (as univariate trait) and shape (as multivariate data) by several authors [87]–[92] was used. This test operates by permuting (10,000 times in our case) the mean values for species (shapes or sizes) to the tips of the phylogenetic tree. Subsequently, ancestral shape reconstructions were recomputed for the permuted data using the squared-change parsimony algorithm of Maddison [93], weighting by branch lengths [86], [87]. A *P*-value indicating the proportion of permutations that result in a tree length equal to or less than our phylogenetic tree inform us on the presence of phylogenetic signal [86], [87]. This *P*-value was used for estimating if phylogenetic structure is present in limb bone shape and size. These tests were performed with software MorphoJ [54].

### Quantifying the influence of size on limb bone shape

The effects of size on interspecific variation in limb bone shape (i.e., interspecific allometry) were tested separately for each bone by multivariate regression analysis [82] of the shape of the analyzed species (i.e., using Procrustes coordinates-Pco) on their size (i.e., using log-transformed Centroid size-Cs). Statistical significance was tested with a permutation test (10,000 in our case) against the null hypothesis of complete size independence [55]. However, given that species cannot be treated as statistically independent entities, as they are related by phylogeny, independent contrast analyses (PIC) [94] for the shape and size of limb bones were performed in order to avoid incorrect interpretations due to a violation of the assumption of independent sampling –i.e., a variant of the classic type I error in statistical analysis [95], [96]. Subsequently, the PIC's for limb bone shapes were regressed on the PIC for limb bone sizes –a multivariate regression through the origin [89], [92]–, which allowed to extract the morphological change between sister nodes of the phylogeny (i.e., evolutionary allometry). The statistical significance of the association between shape PIC's and log-Cs PIC was evaluated with a permutation test against the null hypothesis of complete independence [55]. These multivariate regression analyses were performed with software MorphoJ [54].

### Quantifying the influence of locomotor performance on limb bone shape

In order to test for the association between forelimb morphology and locomotor performance in the living carnivores, two ecological variables were chosen: maximum running speed (MRS) [4], [97], [98] and daily movement distance (DMD) [99], [100]. MRS is a proxy for the hunting skills of carnivores, because it is crucial for the hunting success of many pursuit predators and, consequently, for their survival [3]. Therefore, active predators tend to be faster than those species which rarely hunt –these species do not improve their running speed as it has little effect on their survival–. DMD is a key aspect for locomotor efficiency [99], as those species that travel long distances need to reduce energy expenditures, hence achieving a more efficient locomotion through the increase of their stride length –the highest the more efficient– [13] as well as by constraining the movements of their limbs within the parasagittal plane [15]. Log-transformed values obtained from the literature (see File S1: Table S1) for both MRS (km·h^−1^) and DMD (km per day) were first regressed (OLS) on the Log-Cs of each bone. In order to avoid the possible size effects in subsequent regressions, the residuals were extracted when this correlation was statistically significant. A multivariate regression analysis [82] of each bone shape –the shape residuals obtained from the regression of interspecific allometry– on MRS and DMD (or their residuals) was computed separately for exploring the associations of these variables with shape. Obviously, these analyses were restricted to those living taxa for which data of MRS and DMD were available. A permutation test against the null hypothesis of complete independence of shape on MRS and DMD [55] was used for statistical testing of the association of MRS and DMD with shape. In addition, the PIC for the size-free shape of each limb bone was regressed on the PIC for both log-MRS and log-DMD in order to explore if the association between shape and locomotor performance emerges from phylogenetic patterning. Statistical significance was tested as in the previous multivariate regression. Multivariate regression analyses were performed with software MorphoJ [54].

### Exploring the phenotypic spaces for forelimb bones and their histories of phylogenetic occupation

Given that a principal components analysis (PCA) finds the orthogonal axes of maximum shape variance, it is an appropriate method for exploring the phenotypic variation of limb bones [101]. A PCA was computed separately for each forelimb bone from the covariance matrix for bone shape in the species analyzed, which allowed analyzing the distribution of carnivorans in the phenotypic shape spaces. To explore the shape distribution without the effect of evolutionary allometry, the vector computed from the regression with the independent contrasts of shape and size was applied to the species-averaged dataset [92]. The residuals extracted from this vector, subsequently used for the PCAs, are free of effects from evolutionary allometry [92]. In addition, the phylogenetic history of the occupation of these shape spaces was investigated by reconstructing the ancestral states of limb bone shapes using the squared-change parsimony method weighted by branch lengths [93]. Subsequently, the hypothetical shapes were plotted on the original phenotypic spaces and tree branches were connected to obtain limb bone phylomorphospaces [86]–[91], [102]–[104]. Furthermore, the evolutionary trajectories of shape transformation were explored for each forelimb bone by investigating the shape changes that account from the ancestral states to the tips of the phylogeny. These analyses were performed with software MorphoJ [54].

## Results

### Influence of phylogeny and size on the shape of limb bones

First of all, the multivariate regressions performed between the PIC of the Pco and the standard deviation of the contrasts (SD) yielded non-significant results for the four elements (all the *P*-values were higher than 0.9), which indicates unequivocally that our shape data and our composite tree fit adequately [83] and evidences that the independent contrast analyses were made correctly.

**Figure 3:**
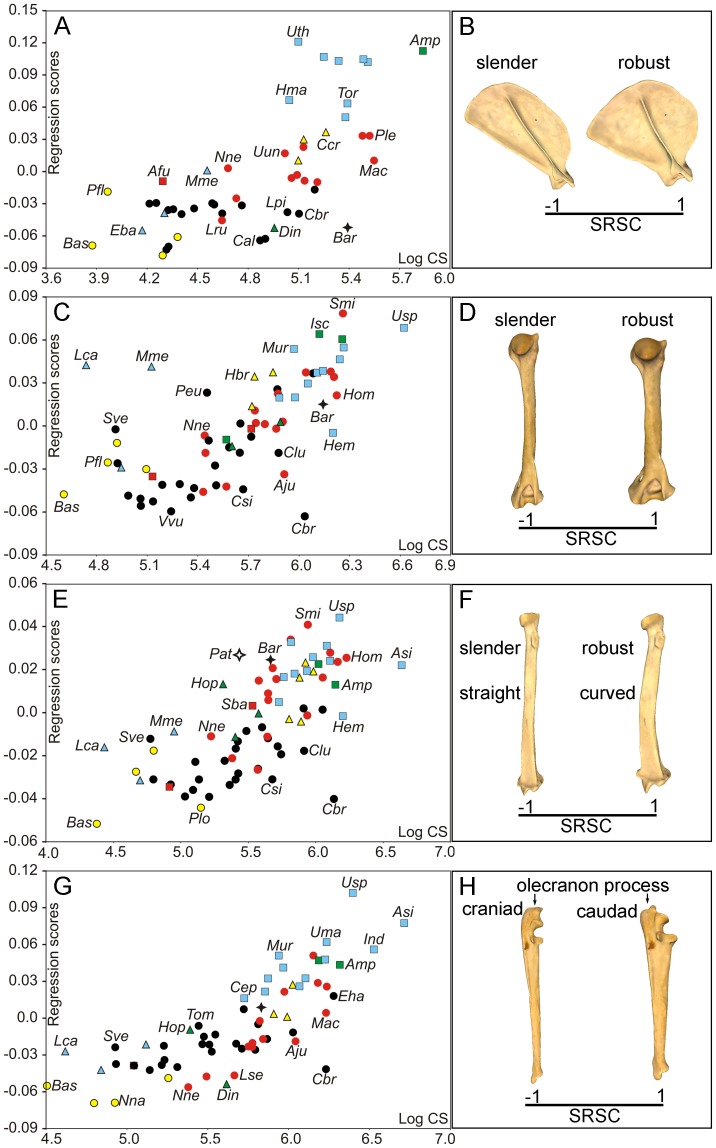
Analysis of interspecific allometry in the carnivoran forelimb. Bivariate graphs for the multivariate regressions performed from Pco against log-Cs values for scapula (A), humerus (C), radius (E) and ulna (G). Three-dimensional models showing size-related shape changes (SRSC) for scapula (B, lateral view), humerus (D, caudal view), radius (F, caudal view) and ulna (H, lateral view) are shown. Symbols: red squares, Ailuridae; green squares, Amphicyonidae; black stars, Barbourofelidae; black circles, Canidae; empty stars, Creodonta; red circles, Felidae; yellow triangles, Hyaenidae; blue triangles, Mustelidae; green triangles, Nimravidae; yellow circles, Procyonidae; blue squares, Ursidae. See file S1: table S1 for species labels. For the interactive three-dimensional shape models explained by the variations in size, see file S3, A–D.

The permutation tests indicated a strong phylogenetic structure for both size and shape of all forelimb bones (Table 1). Multivariate regression analyses of shape (Pco) on size (Cs) performed separately in each forelimb bone for exploring interspecific allometry were highly significant in all cases (Table 2). Figure 3 shows the four multivariate regressions obtained and their associated shape changes. In the proximal limb segments (i.e., scapula and humerus) allometric shape variation is clearly associated with the degree of bone robustness. This means that the scapulae and humeri of small-sized species tend to be more slender than those of large-sized ones, which tend to be noticeably robust (Figure 3A–D; File S3 A, B). However, the distal limb bones (i.e., radius and ulna) show other size-related shape changes in addition to robustness. In the case of the radius, the allometric change is associated with the curvature of the diaphysis: while small radii tend to be slender and more straight, large ones tend to be robust and curved (Figure 3E, F; File S3 C). For the ulna, however, allometry is also associated with the orientation of the olecranon process. As a consequence, small ulnae have a straight olecranon process (aligned with the shaft), while large ones show a caudally oriented olecranon (Figure 3G, H; File S3 D).

**Table 1:** Results obtained for assessing the presence of phylogenetic signal in the shape (Pco) and size (log-Cs) of each forelimb bone.

BONE	*SHAPE*	*SIZE*
**Scapula**	0.249 (<0.0001)	3.6171 (<0.0001)
**Humerus**	0.0933 (<0.0001)	3.8449 (<0.0001)
**Radius**	0.0749 (<0.0001)	5.1574 (<0.0001)
**Ulna**	0.1108 (<0.0001)	4.5214 (<0.0001)

Numbers indicate the tree lengths obtained with each permutation test. *P*-values are provided between brackets.

**Table 2:** Results obtained in the regression analyses.

BONE		*SIZE*	*PIC*	*MRS*	*PIC*	*DMD*	*PIC*
	*SHAPE*	<0.0001 (10.66%)	0.0002	0.066 (10.8%)	0.3743	0.0009 (12.82%)	0.4902
**Scapula**	*MRS*	0.0538 (19.79%)	0.1855				
	*DMD*	0.4929 (1.53%)	0.063				
	*SHAPE*	<0.0001 (16.56%)	<0.0001	<0.0001 (31.44%)	0.3486	0.0077 (10.56%)	0.744
**Humerus**	*MRS*	0.0237 (24.66%)	0.1728				
	*DMD*	0.739 (0.37%)	0.0894				
	*SHAPE*	<0.0001 (10.91%)	0.0025	0.0055 (21.67%)	0.516	0.012 (10.75%)	0.1789
**Radius**	*MRS*	0.0055 (33.36%)	0.1775				
	*DMD*	0.2406 (4.33%)	0.1061				
	*SHAPE*	<0.0001 (17.08%)	<0.0001	0.0077 (24.04%)	0.8819	0.0241 (10.31%)	0.4738
**Ulna**	*MRS*	0.0074 (31.78%)	0.1778				
	*DMD*	0.264 (3.9%)	0.1054				

These regressions were computed from shape (Pco), maximum running speed (log-MRS) and daily movement distance (log-DMD) on size (log-Cs) for each forelimb bone, as well as, the multivariate regression analyses of shape (residuals from the previous regressions) on log-MRS and log-DMD (or their size-free residuals if it was appropriate). The multivariate regression analyses of log-MRS and log-DMD were performed with a restricted dataset according with the availability of data for living species in the literature (see Table S1). Results obtained when phylogenetic independent contrast (PIC) was applied for shape, size, MRS and DMD are also shown. Numbers indicate *p*-values. The percentages of each limb bone shape, log-MRS or log-DMD explained by the differences in the independent variable are given between brackets.

**Figure 4:**
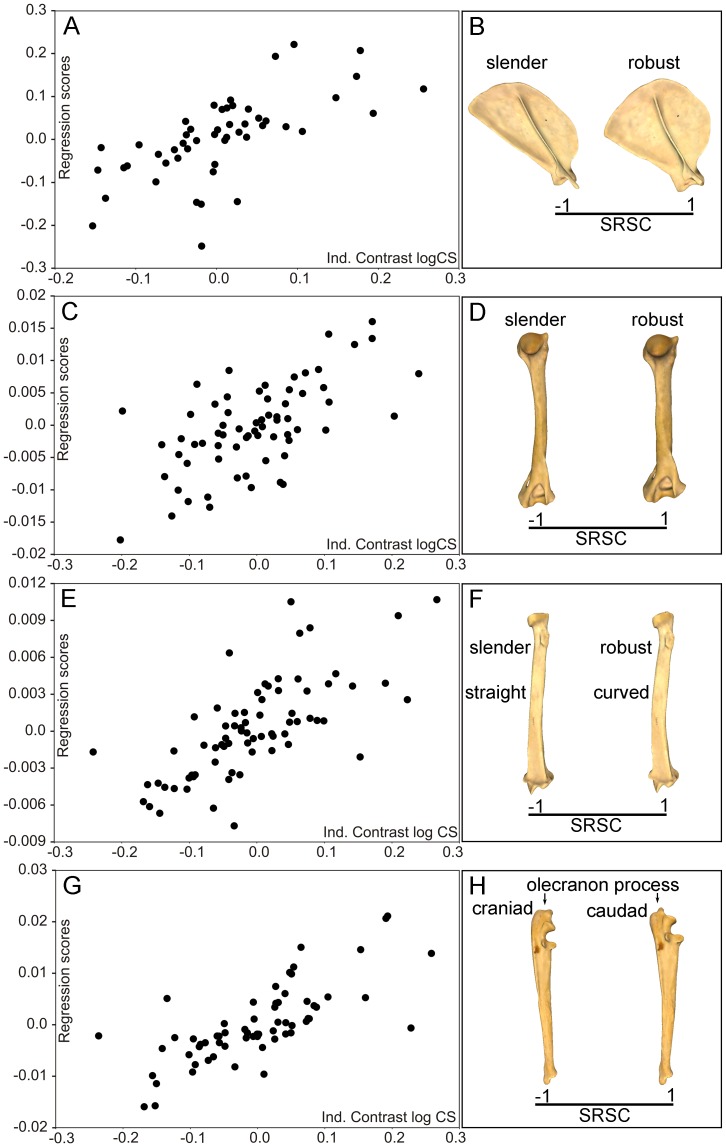
Analysis of evolutionary allometry in the carnivoran forelimb. Multivariate regressions obtained from the PIC Procrustes coordinates (Pco) against the PIC log-centroid size (Cs) and models showing the size-related changes in shape (SRSC) for each bone. Scapula (A, B), humerus (C, D), radius (E, F), ulna (G, H). See file S1: table S1 for species labels.

The multivariate regression analysis performed from the shape PIC against the size PIC (i.e., the one made for testing evolutionary allometry) yielded also highly significant results for the four bones (Table 2). Figure 4 shows the multivariate regressions and their associated shape changes. As in the regression analyses for interspecific allometry, the allometric shape changes obtained for the scapula and the humerus relate with a slight degree of robustness and, in the case of the radius, also with diaphyseal curvature (Figure 4 A–D). However, in the case of the ulna there is not evident change in bone robustness (Figure 4 H). Therefore, size-related shape changes due to evolutionary allometry are only related in the ulna with the orientation of the olecranon process.

Given that a significant influence of size on limb bone shape was detected, the residuals from the multivariate regression of limb bone shape on size were extracted in order to eliminate the predicted component of shape variation due to size differences (see Klingenberg et al. [89]). The residuals of the regression were used as strict shape variables free of allometric effects in the following multivariate regressions.

### Influence of locomotor performance on the shape of limb bones

**Figure 5:**
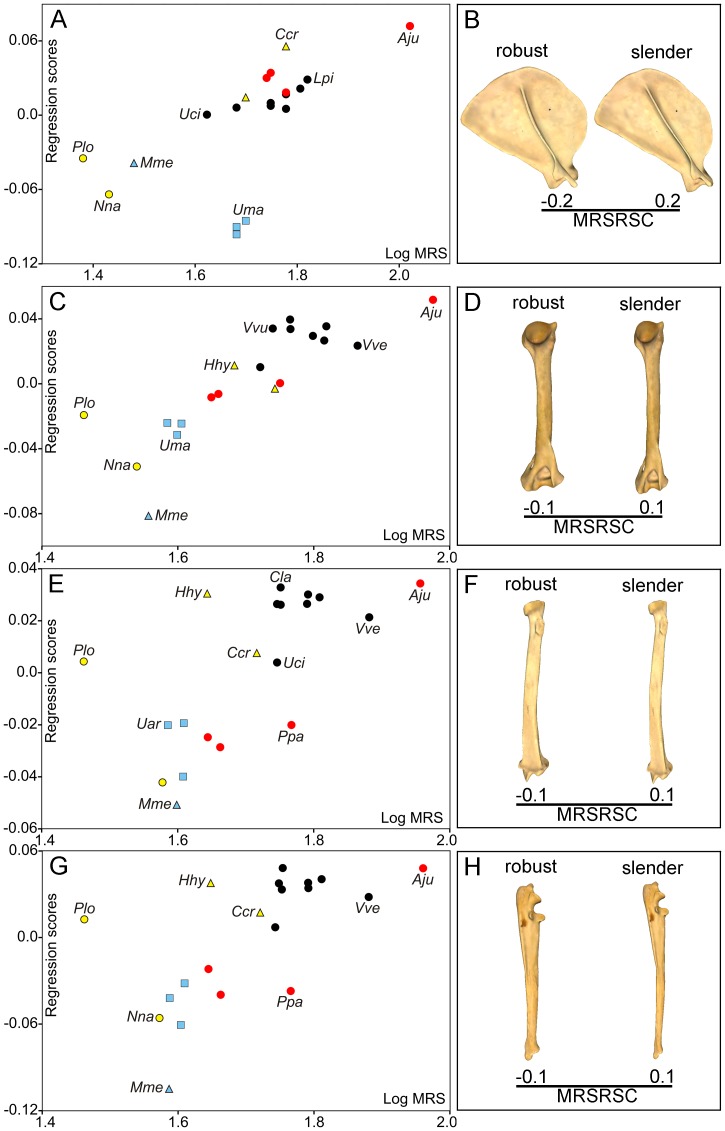
Multivariate regressions of size-free shape against MRS in the carnivoran forelimb. Bivariate graphs depicted from the multivariate regressions performed from the Pco-Cs residuals against the log-MRS values (or their residuals) for scapula (A), humerus (C), radius (E) and ulna (G). Three-dimensional models showing the MRS-related shape changes (MRSRSC) for the scapula (B, lateral view), humerus (D, caudal view), radius (F, caudal view) and ulna (H, lateral view) are also shown. See figure 3 for symbols. See file S1 table S1 for species labels.

Although the regression between the PIC of log-MRS on the PIC of Cs was non-significant (Table 2), the OLS regressions of log-MRS and log-DMD on Cs before the PIC were significant in all bones excepting the scapula (Table 2). Accordingly, the subsequent regressions were performed with the residuals extracted from the OLS regressions of log-MRS on log-Cs for the humerus, radius and ulna. The multivariate regressions between the size-free shape of each forelimb bone and the size-free log-MRS values were statistically significant (Table 2) for all the bones. The four regression analyses and their shape changes are shown in Figure 5. The shape changes associated with MRS are mainly related with bone robustness (Figure 5D). Therefore, while species with low MRS values have robust bones, those with high MRS values show slender ones. In spite of this, multivariate regression analyses performed for the PIC of shape on the PIC of log-MRS were not statistically significant for any bone (Table 2).

**Figure 6:**
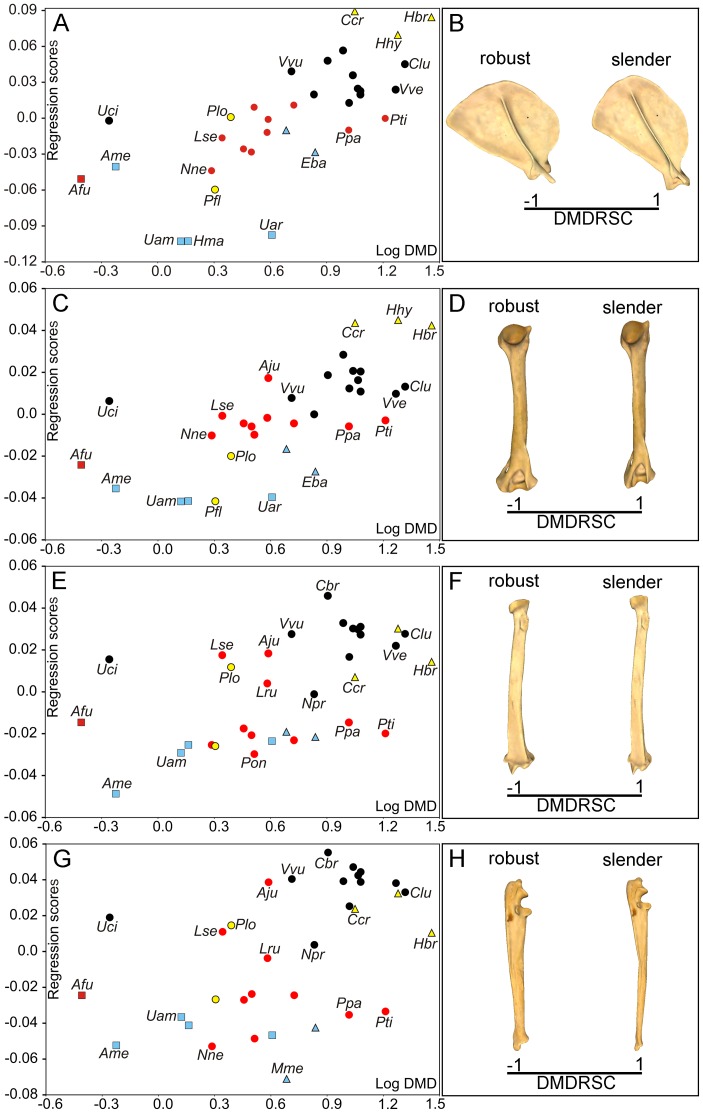
Multivariate regression between shape and DMD in the carnivoran forelimb. Bivariate graphs depicted from the multivariate regressions performed from the Pco-Cs residuals against the log-DMD values for scapula (A), humerus (C), radius (E) and ulna (G). Three-dimensional models showing the DMD-related shape changes (DMDRSC) for the scapula (B, lateral view), humerus (D, caudal view), radius (F, caudal view) and ulna (H, lateral view) are also shown. See figure 3 for symbols. See file S1: table S1 for species labels. For the interactive three-dimensional shape models explained by the variations in DMD, see file S3, E–H.

Given that the OLS regressions of log-DMD on Cs were non-significant for any bone with and without accounting for phylogenetic effects (Table 2), log-DMD values did not need any correction for size effects. Multivariate regressions for the size-free shape of limb bones on log-DMD values were highly significant in all cases (Table 2). With the exception of the ulna, the variation in shape associated to a variation in DMD is mainly explained by a change in bone robustness (Figure 6; File S3 E–H). As a result, the bones from species with low DMD values are more robust than those from species with high DMD values, which tend to be slender. In the case of the ulna, however, low DMD values are associated to a robust and straight anatomy, while high values correspond to a slender and more curved condition (Figure 6H; File S3 H). However, when phylogenetic independent contrast was applied to the regression of shape on log DMD values, an absence of statistical significance was obtained for the four bones (Table 2).

### Limb bone shape spaces and their phylogenetic filling

**Figure 7:**
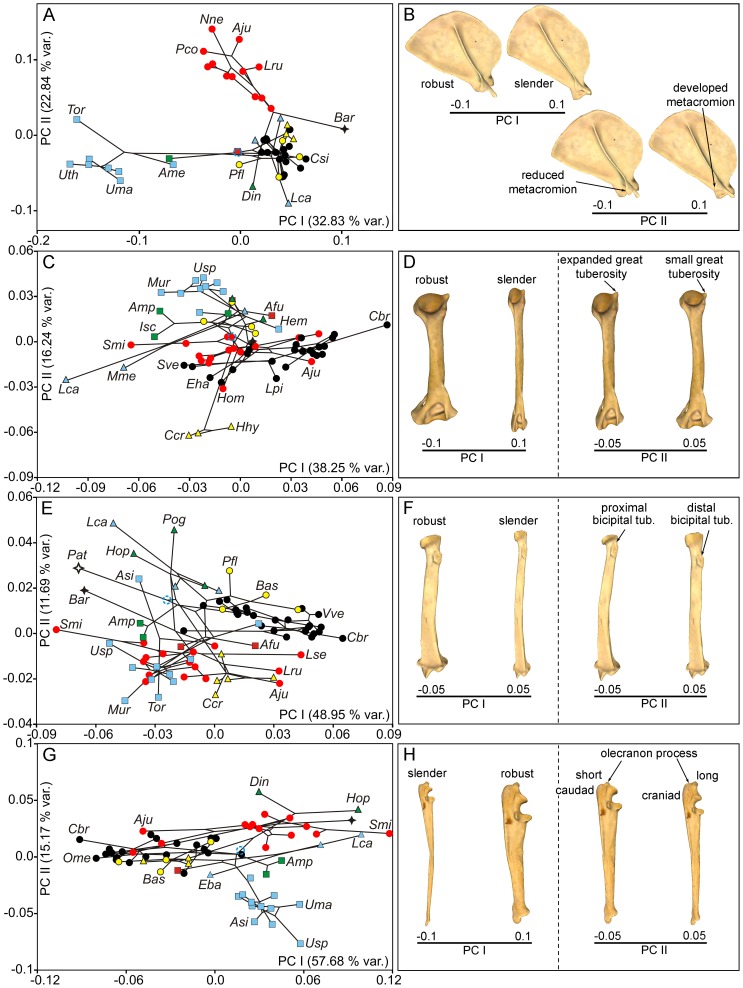
Principal component analyses in the carnivoran forelimb, PC I and PC II. Bivariate graphs depicted from the two first PCs calculated on the PIC regression residuals (see text for details) for scapula (A), humerus (C), radius (E) and ulna (G). Tree topology is also mapped on the morphospaces. Three-dimensional models showing the shape changes associated to these axes for scapula (B, lateral view), humerus (D, caudal view), radius (F, caudal view) and ulna (H, lateral view) are shown. Blue empty circle: tree root; see figure 3 for more symbols. See file S1: table S1 for species labels. For the interactive three-dimensional shape models explained by the PCs, see file S4, A–D.

**Figure 8:**
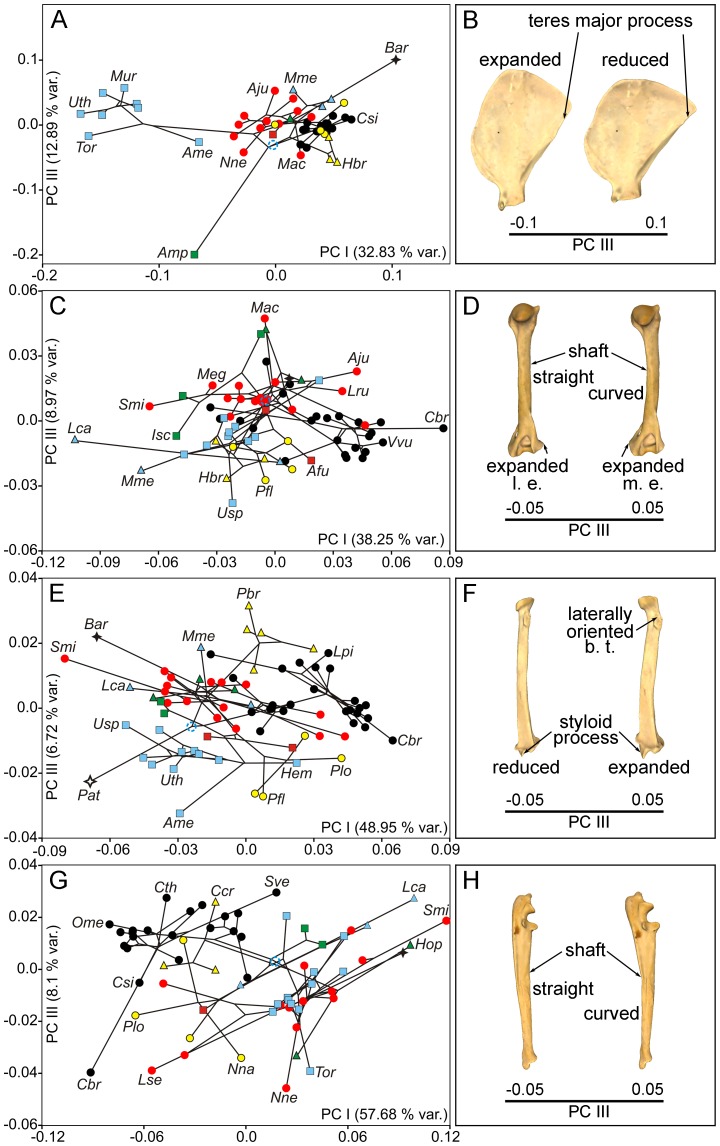
Principal component analyses in the carnivoran forelimb, PC I and PC III. Bivariate graphs depicted from the first and third PCs calculated on the PIC regression residuals (see text for details) for scapula (A), humerus (C), radius (E) and ulna (G). Tree topology is also mapped on the morphospaces. Three-dimensional models showing the shape changes associated to these axes for scapula (B, medial view), humerus (D, caudal view), radius (F, caudal view) and ulna (H, lateral view) are shown. b. t., bicipital tuberosity; l. e., lateral epicondyle; m. e., medial epicondyle. See figure 3 and 7 for symbols. See file S1: table S1 for species labels.

A PCA performed with the covariance matrix for the regression residuals of scapular shape indicated that the first three PCs explained ∼70% of the original variance. Figure 6A shows the phylomorphospace depicted by the first two PCs and the associated shape changes which these eigenvectors account for. The first axis (Figure 7A *x*-axis) mainly depicts the changes from the long and slender scapula of canids and procyonids, which score positively, to the wide and robust one of bears, which shows a well-developed postscapular fossa (which is captured by the landmarks but not by the scanned surface) and take the most negative scores (Figure. 7B; File S4 A). In contrast, the second axis (Figure 7A *y*-axis) relates to the posterior extension of the metacromion process (Figure 7B; File S4 A) and basically describes the changes from the anatomy of felines, which score positively on this eigenvector, to other species included in the sample. The third axis (Figure 8A *y*-axis) is associated with a change in the extension of the teres major process in two extinct taxa: *Barbourofelis* and *Amphicyon*. While *Barbourofelis* scores positively and shows a small teres major process (Figure 8A *y*-axis, B), *Amphicyon* scores negatively with a long process (Figure 8A *y*-axis, B).

Figures 7C and 8C show the phylomorphospaces depicted by PCI on PCII and PCI on PCIII, obtained from the PCA performed on the covariance matrix of the regression residuals of humeral shape, respectively. These eigenvectors account for ∼65% of the original variance. Accordingly, the first PC (Figure. 7C *x*-axis; File S4 B) describes a shape gradient from the slender humeri of the long-legged maned wolf (*Chrysocyon brachyurus*) plus other canine canids, which score positively on this axis, to the robust humeri of the aquatic otter (*Lontra canadensis*) or the Eurasian badger (*Meles meles*), which take the most extreme negative scores (Figure 7D; File S4 B). In contrast, the second PC (Figure 7C *y*-axis. File S4 B) describes the shape change between the expanded greater tuberosity of the humerus in the living hyenas, which score negatively, to the reduced greater tuberosity of the humerus in all living bears, which take positive scores (Figure 7D; File S4 B). The third PC (Figure 8C *y*-axis, D) accounts for the curvature of the humeral shaft and also for the relative expansion of the two epicondyles of the distal epiphysis. Those species with positive scores –e.g., *Machairodus*, *Nimravus* and *Daphoenus*– have a laterally curved shaft and an expanded medial epicondyle (Figure 8C *y*-axis, D). In contrast, those species which score negatively –*Ursus spelaeus* and some procyonids, mustelids and hyaenids– have a straight humeral shaft and an expanded lateral epicondyle (Figure 8C, D).

In the case of the PCA performed on the covariance matrix of the regression residuals of radius shape, the first three PCs explained ∼67% of the original variance. Figure 7E shows the phylomorphospace depicted by these first two eigenvectors as well as the morphological changes that they account for. The first axis mainly describes the change from the slender radius of most canids, particularly evidenced in the long-legged *Chrysocyon brachyurus*, which take extreme positive scores (Figure 7E *x*-axis, F; File S4 C), to the robust radii of *Lontra canadensis*, the cave bear *Ursus spelaeus*, the Pleistocene saber-tooth *Smilodon*, the creodont *Patriofelis*, and the false-sabertooth *Barbourofelis*, which all score negatively. In contrast, the second axis depicts a change in the position of the bicipital tuberosity (Figure 7F *y*-axis; File S4 C). The sloth bear *Melursus ursinus* has this tuberosity more proximally placed (i.e., it is expanded towards the proximal epiphysis of the radius) and scores with extreme negative values. In contrast, the nimravid *Pogonodon* sp. and the Canadian river otter *Lontra canadensis* have both a distally positioned (and more reduced) bicipital tuberosity, showing positive scores (Figure 7E *y-axis*, F; File S4 C). The third PC distributes the species according to the position of the bicipital tuberosity and the expansion of the styloid process (Figure 8E *y*-axis, F). The positive scores of hyaenids, *Barbourofelis* and *Smilodon* indicate a more lateral orientation of the bicipital tuberosity and an expanded styloid process (Figure 8E *y*-axis, F). In contrast, the negative scores of ursids, procyonids and *Patriofelis* result from a posterior orientation of the bicipital tuberosity and a reduced styloid process (Figure 8E *y*-axis, F).

Figures 7G and 8G show the phylomorphospaces depicted by PCI on PCII and the PCI on PCIII, derived from the PCA performed on the covariance matrix of the regression residuals of ulnar shape, respectively. The morphological changes accounted for by these eigenvectors, which jointly explain ∼81% of the original variance, are depicted in Figure 7H. The first eigenvector describes the morphological change from the robust ulna of the saber-tooth *Smilodon*, which scores positively, to the slender ulna of the maned wolf (*Chrysocyon. brachyurus*), which takes negative scores (Figure 7G x-axis, H; File S4 D). In contrast, the second eigenvector accounts for the morphological gradient related with the length and orientation of the olecranon process. While all living bears score negatively on this axis, which results from their short and caudally oriented olecranon, most felid-like carnivores score positively, which evidences the presence of a long olecranon, which is aligned with the shaft (Figure 7G y-axis, H; File S4 D). The third PC depicts a change in curvature of the ulnar shaft (Figure 8G y-axis, H). Positive scores on this axis correspond to an antero-posteriorly curved shaft, typical of most canids and other species like *Crocuta crocuta*, *Lontra canadensis* and *Smilodon* (Figure 8G y-axis, H). Those species with negative scores –*Chrysocyon brachyurus*, *Leptailurus serval*, *Neofelis nebulosa*, *Tremarctos ornatus* and *Nasua nasua*, among others– have a straight ulnar shaft (Figure 8G y-axis, H).

### Phylogenetic patterns in limb bone shape spaces and ancestral bone reconstruction

**Figure 9:**
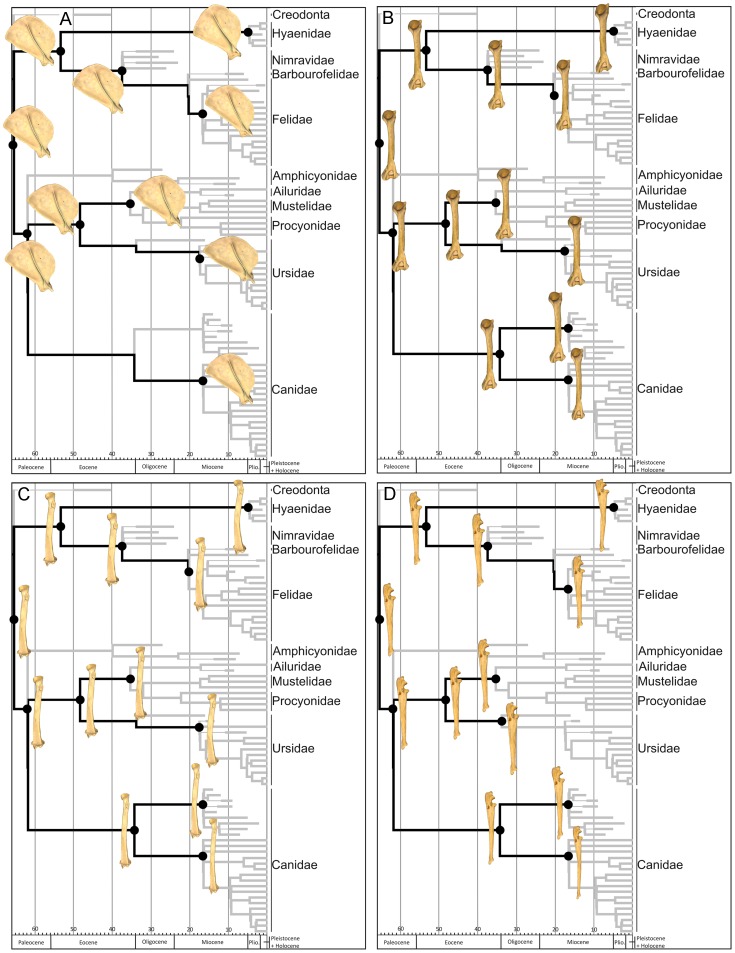
Reconstruction of ancestral forelimb bone shapes. Scapula (A), humerus (B), radius (C), ulna (D). Three-dimensional models show hypothetical morphologies for the nodes highlighted (black circles).

Visual inspection of the phylomorphospace depicted from scapular shape in Figures 7A and 8A shows that although many terminal branches of the tree are relatively short, the internal branches are longer. This is also applied to *Barbourofelis* and *Amphicyon* in Figure 8A, because despite that they are terminal branches, they also represent different families. This suggests that closely related species have similar scapular shapes and, as a consequence, the different carnivoran families are confined to well differentiated portions of the shape space (Figures 7A and 8A). This indicates that phylogenetic legacy had a strong influence on the evolution of scapular morphology, which is highly conservative within families. In fact, the reconstructed ancestral states for the internal nodes of the phylogenetic tree (Figure 2) indicate that the ancestors from each carnivoran family all showed different scapular shapes (Figure 9A) and that these shapes were maintained during their evolution (i.e., the taxa that belong to the same family experienced few changes in scapular morphology). Strikingly, the phylomorphospaces depicted from the shape of three major long bones (i.e., humerus, radius and ulna) are clearly more “messy” than the one performed from the scapula (compare Figure 7A and 8A with Figures 7C, E, G and 8C, E, G), indicated by the presence of many long terminal branches and relatively short internal branches. This pattern suggests a high level of homoplasy [86], [87]. In fact, the reconstructed ancestral states for these three bones (Figure 9B, C, D) clearly indicate that although the basal family nodes are different, these shapes were not maintained through the evolution of those taxa that belong to the same family. In contrast, other potential aspects influence the shape of these long bones and override to some extent their phylogenetic structure. This difference may be explained if we consider that the scapula, which is part of the shoulder girdle, has a different origin and evolutionary history compared to the long bones [105]. Furthermore, there may be also structural reasons, because the long bones are more exposed than the scapula to changes in robustness due to their particular shape and structural position within the forelimb.

## Discussion

### Phylogeny and size as the main sources of bone shape variation

According to the permutation test, there is a strong phylogenetic signal in the shape and size of all forelimb bones. This indicates that evolutionary changes in both size and shape in the bones of the forelimb are highly conservative within the order Carnivora. Furthermore, as the species included in our analyses cover a wide range of size and morphology in both the living and extinct taxa, these results are not likely to be an artefact from sampling bias. In contrast, we hypothesize that the morphology and size of the main forelimb bones were acquired early in the typical “bauplan” of each carnivoran family. This result is not surprising, as many authors have found similar significant results for several taxa [9], [23], [36]. It is worth noting, however, that collecting more data for some families with a high variability in locomotor modes (e.g., mustelids) may give additional information about the effect of the phylogenetic structure in the evolution of forelimb shape and size. Furthermore, we have only tested here the evolution of size under the model of Brownian motion. Future research for testing other models of size evolution (e.g., Ornstein-Uhlenbeck) could add new information on the possible trends in size change followed during the evolution of the different forelimb bones.

In agreement with previous studies [3], [32], [35], [106], [107], our results demonstrate that the morphology of the forelimb bones is highly influenced by variations in size. This significant association does not merely result from a secondary correlation in the phylogeny, as indicated by phylogenetic independent contrast. With the only exception of the radius and ulna, size-related shape changes are mainly associated with the degree of bone robustness. Allometric shape change relates in the radius to the curvature of the diaphyseal shaft. In the ulna there is a change in the orientation of the olecranon process.

Therefore, our results agree with the prediction that larger animals need more robust limb bones for resisting the increase in stress resulting from increased body mass [106]. However, other scaling studies in mammalian limb bones [108], [109] have demonstrated that no general scaling rule applies, because there are alternative mechanisms for reducing the peak stresses on the bones, for example to increase the effective mechanical advantage by adopting a more upright posture [108], [109]. However, it is worth mentioning that Day and Jayne [110] found compelling evidence for the absence of a relationship in the family Felidae between changes in posture, on the one hand, and both size and walking gait, on the other. Our results indicate that allometric shape changes relate in the ulna with changes in body posture. Furthermore, the absence in this bone of changes in robustness associated to size variations probably reflects that it is marginally connected to the wrist (i.e., the carpals) and most of the ground reaction force is transmitted through the radius shaft. The case of the radius is also striking, because while larger animals have more robust radii than smaller ones, they also have a more curved diaphyseal shaft. This could be also associated with the necessity to withstand greater stresses, as this curvature may be linked with the fact that the distal epiphysis increases in robustness with size more than the proximal one.

### Locomotor performance as a not significant source of shape variation

A good correlation between shape and MRS was obtained for each forelimb bone. However, this correlation disappears when phylogenetic independent contrast is applied. There are two possible causes that could explain the absence of correlation between shape and MRS. The first is that MRS does not affect the shape of the forelimb bones. The second explanation, however, could result from the fact that reliable MRS data of carnivores are scarce in the literature (only 20 species) and this scarcity could be hiding the actual correlation. Furthermore, it is worth mentioning that MRS data may be biased towards the large terrestrial carnivorans, as MRS data for arboreal or burrowing species are comparatively scarce (see File S1: Table S1). In spite of this, previous research with larger samples that include other mammalian orders and using conventional metric variables have shown only modest correlations between forelimb shape and MRS [4], [98].

Similarly to the case of MRS, all bone shapes correlate with daily movement distance (DMD), showing a morphological change associated with bone robustness. Those species with higher DMD values have more slender bones and *vice versa*. This is in agreement with the widespread thought that longer and slender limbs increase locomotor efficiency [13], [111]. However, this correlation vanished when phylogenetic independent contrast was applied to our data. Therefore, our results indicate that both DMD and bone shape are strongly influenced by phylogenetic pattering, because those carnivores from the same family have a restricted range of DMD values but these aspects vary independently within each family.

MRS and DMD are two widespread used proxies for hunting styles and running abilities of predators, respectively. Given that our data suggest that the shape of the forelimb bones is not indicative of those aspects of locomotion recovered by both MRS and DMD, these results are in part counter intuitive, as one of the main functions of the appendicular skeleton is locomotor performance (but see Harris and Steudel [100]). In any case, inspection of the distribution of taxa in the phylomorphospaces for the four major forelimb bones may provide insights on such unexpected results. However, we must emphasize that this does not mean that bone morphology does not reflect functional adaptations, as many other aspects of locomotor behavior (e.g., climbing, burrowing or swimming) are not directly related to MRS and DMD. Furthermore, although for many carnivorans included in this study MRS has little impact on whether or not these predators can acquire their prey (e.g., mustelids and procyonids), this is actually reflected in their low values for this variable (see Table S1).

### Phylomorphospaces and morphological variability

The separate distribution of carnivoran families in the scapular shape spaces suggests a strong influence of phylogenetic legacy on the evolution of this bone. Furthermore, the reconstructed ancestral states for scapular morphology indicate that these states were acquired early in the evolution of each family and, once acquired, they remained constant among taxa within families. In contrast, the shapes of the three long bones show more overlap between the families in their respective phylomorphospaces, as indicated by a more “messy” appearance, which suggests higher levels of homoplasy. As a result, it may be deduced that the influence of functional factors on these bones could be overriding the phylogenetic legacy of each family, at least more than in the case of the scapula. In fact, the first PC's for these three bones arrange the species according to their degree of bone robustness (see Figure 7), showing that distantly related taxa may exhibit similar morphologies. For example, on the one hand, a slender anatomy for humerus, radius and ulna is present in most canine canids (with the manned wolf, *Chrysocyon brachyurus*, as the most extreme example), hyaenids, the cheetah (*Acinonyx jubatus*), the bobcat (*Lynx rufus*) and the serval (*Leptailurus serval*) among the felids, the extinct “dog-like” bear *Hemicyon* among the ursids, and the racoon (*Procyon lotor*) among the procyonids. A slender condition has been usually interpreted as an adaptation of long bones to reduce the energetic costs of terrestrial locomotion by decreasing the moment of inertia of limbs and by increasing stride length [13], [32], [112]. Therefore, to have slender long bones allows the animal to travel longer distances on the ground (by increasing stamina) and/or to run faster. In other words, this adaptation is related with cursoriality [12], [13], [26], [32], [113], [114].

However, there are multiple ecological scenarios where this morphological trait can enhance fitness [13], [113]. For instance, it is clear that this morphology represents an adaptation for the cheetah to increase speed during active pursuit of prey [113], [115]. In contrast, the slender long bones of most pack-hunting canids and hyenids mainly reflect their adaptation towards increasing stamina during long distance foraging [13], [99], [116]. In the case of the serval, the scenario is quite different as its slender forelimb bones most probably represent an adaptation for walking over the tall grasses typical of its habitat [13], [110]. The racoon also has a slender forelimb, which probably reflects an adaptation for its special manipulatory abilities [117].

On the other hand, there are distantly related species with extremely robust forelimb bones (see Figure 7). This is the case of the Canadian river otter (*Lontra canadensis*) and the European badger (*Meles meles*) among the mustelids, the saber-tooth cat *Smilodon* sp. and the “false” saber-tooths *Barbourofelis* sp. and *Hoplophoneus* sp., among cats and “cat-like” species, and *Patriofelis* sp. for the extinct order Creodonta. The case of the bush dog (*Speothos venaticus*) is also interesting, as this hypercarnivorous canid diverges from other canine canids towards more robust limb bones, although it does not reach the level of the species cited above. The presence of robust forelimb bones has been traditionally interpreted as an adaptation for resisting axial and bending stresses [14]. However, such stresses can result from different activities depending on specific life styles. For example, the robust forelimb of saber-tooths has been previously proposed by other authors to be an adaptation to grasp and subdue their prey to perform a quick and highly specialized killing bite in the throat [12], [14], [22], [26], because their highly elongated and laterally compressed canines precluded these predators to use them for immobilizing their prey [118]. However, in other taxa the possession of a robust forelimb may represent different adaptations depending upon their ecological strategies. For example, the robust forelimb of the otter represents an undisputable adaptation to swim [113] which could be also the case of the bush dog, which has a semiaquiatic life style and shows webbed feet [119], [120]. *Patriofelis* sp. also has a robust radius. Although it is worth noting that this species was initially thought to be aquatic or semi-aquatic [121], Osborn [122] established a felid-like behavior rather than a semi-aquatic life style for this taxon. In the case of the European badger, however, its robust forelimb reflects an adaptation to dig burrows [12], [18], [113].

In summary, species with different ecologies but similar biomechanical needs share parallel morphological changes towards limited zones of the morphospace (i.e., robust vs. slender bones). According to the results obtained in PCA, we hypothesize that, after accounting for size differences, the main factor shaping forelimb bones was a trade-off between the need of powerful bones for resisting different kinds of stresses and the need of reducing the cost of locomotion by having a slender forelimb, as was previously proposed for dog breeds by Carrier and colleagues [123], [124]. However, as noted above, there are many ecological and behavioral contexts which could favour one or another biomechanical and/or morphological solution. As a consequence, there are several morphological convergences among species with quite different ecological habits. Most probably, the results obtained in PCA could explain why there is a poor correlation between forelimb bone shape and both proxies for locomotor performance (i.e., MRS and DMD). However, we must take into account the importance of considering all bones together, particularly in those cases where the relative proportions of elements become more important for interpreting locomotor performance. For example, both semi-aquatic carnivorans and rodents show relatively short and robust femora, which are paired with a relatively elongated tibia and enlarged metatarsals [7], [26]. The result is that if these elements are considered separately, it could lead to incorrect interpretations.

Although the main variance in shape obtained with the PCA performed with all forelimb bones –i.e., the variance accounted for by the first PC– is related with changes in the degree of robustness described above, the second and third PCs account for morphological changes with other biomechanical implications. The reason is straightforward, as these morphological features indicate different functional adaptations related to different biomechanical needs. The importance of some muscles for limb join mobility is clearly reflected in these morphological changes. For example, the second PC obtained from the shape of the scapula accounts for changes in the expansion of the acromion and metacromion processes (see Figure 7A, B). Accordingly, felids have both processes more expanded (see Figure 7A, B), which could reflect an emphasized function for the acromiodeltoid muscle, which originates from them [51]. This fact could be associated with the reported counteraction of the ground reaction forces during locomotion in this family [125]. In contrast, the third component obtained from scapula shape distinguishes the expansion of the teres major process in two extinct taxa: *Amphicyon* –with an expanded process– and *Barbourofelis* –with a reduced process– (see Figure 8A, B), which probably indicates more emphasis on the function performed by this muscle in the first taxa. However, to derive ecological interpretations here is too speculative, as there is an absence of close living relatives and ecological analogues for both extinct taxa.

The second component of humeral variability accounts for a change in the greater tuberosity (see Figure 7D). This tuberosity represents the insertion of the supraspinatus muscle (see Figure 1), which mainly function is to protract the humerus [51]. Although this trait shows a strong phylogenetic signal (see Figure 7C), it also has biomechanical implications, because the moment arm for the supraspinatus muscle indicates its relevance during locomotion. In those species with a larger moment arm (e.g., felids, canids and, especially, hyaenids [126]), this muscle plays an important role during galloping. The reason is that the supraspinatus muscle assists other muscles in the extension phase of the stance, at least in the case of canids [127]. In contrast, the species with a smaller moment arm (e.g., ursids, ailurids and procyonids) rarely gallop, so the function of this muscle in them is to stabilize the shoulder joint [127].

We hypothesize that the changes in the curvature of the humeral shaft and in the expansion of the distal epicondyles accounted for by the third PC (see Figure 8D) may be related to changes in animal posture (i.e., adducted or abducted forelimbs). The expansion of the medial epicondyle is associated with an increased mechanical advantage of the pronator teres muscle and of the digital flexor muscles [50]. In addition, a laterally curved humeral shaft indicates an adducted position for the humerus. Accordingly, the humeral shaft remains vertical to transmit the axial loads with a more pronated forepaw. However, we urge readers to interpret this with caution, because the three taxa that show this peculiar morphology, which score positively on PCIII, are extinct –*Machairodus*, *Nimravus* and *Daphoenus* (see Figure 8C; *y*-*axis*). Although in a lesser degree, felids and borophagines also have an expanded medial epicondyle, which may suggest a similarity in the function of the forepaw between these two groups. Nevertheless, this morphological coincidence does not imply necessarily an ecological convergence, as several muscles and strucutures are involved. In the opposite extreme of PCIII (i.e., those taxa with negative scores), the humeri have a straight shaft and an expanded lateral epicondyle, which both indicate a more vertical position for this bone and an increased ability to supinate the forearm. This morphology is common to procyonids, hyaenids, some ursids and canines. Therefore, due to the ecological variability of these taxa, the expansion of the lateral epicondyle is also difficult to interpret from an ecological point of view.

The PCA performed from radius shape indicate that the second and third eigenvectors account for changes in the bicipital tuberosity of the radius (Figure 7F; Figure 8F), which reflects the forces exerted by the biceps brachii muscle. It is worth noting that this muscle is inserted in the bicipital tuberosity (Figure 1). Accordingly, felids and ursids have a more proximal and posteriorly positioned bicipital tuberosity, which indicates a more important role of the biceps brachii in the forearm movements. In contrast, fully terrestrial canids or hyaenids have a more distal and/or lateral position of the bicipital tuberosity, which suggests a minor role of the biceps brachii to perform forearm movements. Given that the position of bicipital tuberosity along both the proximo-distal and latero-medial axes is not related with the insertion area of the biceps brachii, there is not an unequivocal link between the position of the former and the development of the later. The third component shows also an expansion of the styloid process in several taxa analyzed, mainly hyaenids (Figure 8E, F), which points towards a reduced mediolateral mobility of the forepaw in this group. In contrast, a reduced styloid process provides higher mobility to the wrist, which is likely associated with the manipulating and climbing abilities of ursids and procyonids, as they occupy this area of the morphospace (Figure 8E, F).

The change in the length and orientation of the olecranon process of the ulna, depicted by PCII, implies a variation of the moment arm for the major forearm extensor, the triceps brachii muscle (Figure 1). For example, the triceps brachii muscle is less powerful when the olecranon is short and caudally oriented. However, with a long and cranially oriented olecranon, the triceps brachii muscle is more powerful and functions with a more flexed forearm. These shape differences would indicate different locomotor abilities. Accordingly, ursids show a short and caudally oriented olecranon (Figure 7G, H) and, therefore, have a more upright posture and less mechanical advantage of the triceps brachii muscle. Canids and hyaenids have a moderately long and caudally oriented olecranon process (Figure 7G, H), a trait which, together with their slender ulna, is characteristic of fully terrestrial carnivores [12]. Cats and “cat-like” species (i.e., barbourofelids and nimravids) have a longer and more cranially oriented olecranon, which is associated with their hunting strategy: modern felids use their forearms for manipulating and subduing their prey before the killing bite [12], [14], [22], [26], thus needing a powerful triceps brachii to exert enough force. The position of the forelimb in these situations is usually more flexed than in the normal standing posture and, probably for this reason, the olecranon is cranially oriented.

The third PC shows a morphological change associated with a posterior curvature of the ulnar shaft (see Figure 8H), which allows a more upright posture for the limb while keeping constant the orientation of the olecranon and, thus, the mechanical advantage for the triceps brachii muscle. Therefore, these morphological changes are associated with a different way to increase the mechanical advantage for the triceps brachii when the forearm is extended than the one showed by PCII. The distribution of species along this axis (Figure 8G) indicates that some of them belonging to different groups have achieved the curved ulna independently –*Speothos venaticus*, *Crocuta crocuta*, *Lontra canadensis*–, and probably in response to different ecological contexts. The species placed in the opposite extreme –*Chrysocyon brachyurus*, *Neofelis nebulosa*, *Leptailurus serval*, *Tremarctos ornatus*– also suggest an independent evolution under different ecological scenarios.

## Conclusions

The most important bone elements of the forelimb represent a remarkable case of evolution in which similar morphologies were acquired to afford the same biomechanical necessities generated in extremely different ecological scenarios. The results of PCA can be interpreted as evidencing that most of the morphological variation in all these bones is associated with two morphological solutions (slender vs. robust) for affording several ecological problems. For example, robust forelimb bones evolved towards manipulating large prey (as in the case of saber-tooths), towards digging (in the case of the European badger), or for maneuvering during swimming (in the case of the Canadian river otter). Therefore, different ecological requirements can be solved with a single morphology, in this case having robust bones, as they have similar biomechanical demands. In contrast, slender bones evolved towards fast-running (in the case of the cheetah) or to travel longer distances by increasing stamina (in the case of foxes or wolves). Again, the same morphological solution (slender limbs) addresses different ecological problems with similar biomechanical demands. So, we hypothesize that the morphology of the forelimb bones is constrained towards being slender and robust. With only these two morphological extremes, different unrelated taxa have been adapted to afford the same biomechanical requirements in different ecological scenarios. Therefore, the “general morphology” of the forelimb bones represents an extreme “one-to-many mapping” case with a remarkable absence of specific morphological convergences towards the same ecological environments. We propose that this absence of a high phenotypic variability in the forelimb bones is probably associated to balance a trade-off between maintaining resistance to stresses and increasing the energetic efficiency of locomotion. As a consequence, those species in which resistance to different stresses was enhanced at the expense of reducing locomotion efficiency evolved towards having more robust forelimb bones. In contrast, those species that have a more efficient locomotion evolved towards a more slender condition. However, there are many environmental regimes (or ecologies) in which one or another morphological solution could be favoured by natural selection. This “one-to-many mapping” case between shape and ecology led to an absence of correlation between the shape of the forelimb bones and both MRS and DMD.

In spite of these, some morphological changes indicative of specific adaptations to improve the function of the forelimb through the modification of muscle mechanical advantages and joint mobility are also recognized in PCA. However, these functional adaptations are very diverse and they hardly reflect ecology, as one trait could be useful for different activities. Therefore, we will use in future projects other analyses such us canonical variates analysis or linear discriminant functions in the search for specific bone shape features that finely reflect functional adaptations in carnivorans –instead of PCA, which allows describing the major axes of shape variation. Accordingly, although in this paper we quantify the general morphology of the forelimb bones and its meaning, future research will be conducted to find those bone features that better reflect specific functional adaptations.

## Supporting Information

File S1 **TABLE S1.** Sample sizes used in this study for each forelimb bone (S, scapula; H, humerus; R, radius; U, ulna). Log-transformed values of daily movement distance (DMD, km per day) and maximal running speed (MRS, km·h^−1^) for those taxa with available data in the literature are also shown. Daggers (†) represents extinct species. Numbers indicate source references. TABLE S2. List of specimens for the living species included in this paper. Host institution and identity number (ID) are indicated. AMNH, American Museum of Natural History (New York); NHM, Natural History Museum (London). * Indicates a specimen in which the scapula was absent; the four bones analyzed here were present for the remaining specimens. TABLE S3. List of fossil specimens included in this paper. Host institution and identity number (ID) are indicated. AMNH, American Museum of Natural History (New York); NHM, Natural History Museum (London); NMB, Naturhistorisches Museum (Basel); MNCN, Museo Nacional de Ciencias Naturales (Madrid); MSN, Museo di Storia Naturale (Firenze); SNM, Staten Naturhistoriske Museum (Copenhagen); MCNV, Museo de Ciencias Naturales de Valencia (Valencia). TABLE S4. Detailed description of the anatomical position of each landmark used in this study. TABLE S5. Stratigraphic ranges and time of divergence for the extinct taxa included in the composite tree used in this paper. The source references for phylogenetic position and stratigraphic range are indicated. The time of divergence of two extinct species (*Arctodus simus* and *Ursus spelaeus*) have been obtained from molecular data (MD). The time of divergence for the order Creodonta and the family Amphicyonidae was conditioned by the times of divergence given by Nyakatura and Bininda-Emonds (2012) for the living Carnivora. References are listed in the main text.
(DOC)

Figure S1 **Detailed figure of the landmarks used in this paper.**
(TIF)

File S2 **Nexus file of the composite tree used in this paper.**
(PDF)

File S3 **Interactive three-dimensional models of shape variation in the carnivoran forelimb.** Size-related shape changes for scapula (A), humerus (B), radius (C) and ulna (D). Shape changes accounted for by variation in DMD in the scapula (E), humerus (F), radius (G) and ulna (H). Left indicates negative regression scores and right positive ones.
(PDF)

File S4 **Three-dimensional models showing the shape changes obtained from the PCA's.** A, scapula; B, humerus; C, radius; D, ulna. PC I top, PC II bottom; left for negative scores, right for positive scores.
(PDF)
