# Supplementary material for: A Three-Dimensional Analysis of Morphological Evolution and Locomotor Performance of the Carnivoran Forelimb
Source: PLoS One. 2014 Jan 15;9(1):e85574. doi: 10.1371/journal.pone.0085574 (PMC3893248; doi:10.1371/journal.pone.0085574)
Supplement: File S1 — TABLE S1. Sample sizes used in this study for each forelimb bone (S, scapula; H, humerus; R, radius; U, ulna). Log-transformed values of daily movement distance (DMD, km per day) and maximal running speed (MRS, km·h−1) for those taxa with available data in the literature are also shown. Daggers (†) represents extinct species. Numbers indicate source references. TABLE S2. List of specimens for the living species included in this paper. Host institution and identity number (ID) are indicated. AMNH, American Museum of Natural History (New York); NHM, Natural History Museum (London). * Indicates a specimen in which the scapula was absent; the four bones analyzed here were present for the remaining specimens. TABLE S3. List of fossil specimens included in this paper. Host institution and identity number (ID) are indicated. AMNH, American Museum of Natural History (New York); NHM, Natural History Museum (London); NMB, Naturhistorisches Museum (Basel); MNCN, Museo Nacional de Ciencias Naturales (Madrid); MSN, Museo di Storia Naturale (Firenze); SNM, Staten Naturhistoriske Museum (Copenhagen); MCNV, Museo de Ciencias Naturales de Valencia (Valencia). TABLE S4. Detailed description of the anatomical position of each landmark used in this study. TABLE S5. Stratigraphic ranges and time of divergence for the extinct taxa included in the composite tree used in this paper. The source references for phylogenetic position and stratigraphic range are indicated. The time of divergence of two extinct species (Arctodus simus and Ursus spelaeus) have been obtained from molecular data (MD). The time of divergence for the order Creodonta and the family Amphicyonidae was conditioned by the times of divergence given by Nyakatura and Bininda-Emonds (2012) for the living Carnivora. References are listed in the main text. (DOC) [file pone.0085574.s001.doc]

**FILE S1**

TABLE S1.

| **Family** | **Species (abbreviation)** | **S** | **H** | **R** | **U** | **DMD** | **MRS** |
| --- | --- | --- | --- | --- | --- | --- | --- |
| **Ailuridae** | *Ailurus fulgens (Afu)* | 2 | 2 | 2 | 2 | 0.41^1,2,3^ | - |
|  | *Simocyon batalleri (Sba)* † | 0 | 2 | 2 | 0 | - | - |
| **Amphicyonidae** | *Amphicyon* sp. (*Amp)* † | 1 | 6 | 14 | 11 | - | - |
|  | *Daphoenus* sp. *(Dap)* † | 0 | 1 | 0 | 0 | - | - |
|  | *Ischyrocyon* sp. *(Isc)* † | 0 | 2 | 3 | 2 | - | - |
| **Barbourofelidae** | *Barbourofelis* sp. *(Bar)* † | 1 | 1 | 5 | 2 | - | - |
| **Canidae** | *Aelurodon ferox (Afe)* † | 0 | 1 | 5 | 2 | - | - |
|  | *Aelurodon taxoides (Ata)* † | 0 | 3 | 2 | 2 | - | - |
|  | *Borophagus* sp. *(Bor)* † | 0 | 0 | 3 | 0 | - | - |
|  | *Canis adustus (Cad)* | 1 | 1 | 1 | 1 | - | - |
|  | *Canis aureus (Cau)* | 2 | 2 | 2 | 2 | 1.08^4^ | 1.75^43^ |
|  | *Canis latrans (Cla)* | 5 | 5 | 5 | 5 | 1.04^5, 6, 7, 8^ | 1.75^43^ |
|  | *Canis lupus (Clu)* | 5 | 5 | 5 | 5 | 1.32^5, 9, 10, 11^ | 1.81^43^ |
|  | *Canis mesomelas (Cme)* | 4 | 4 | 4 | 4 | 1.07^12,13^ | 1.78^43^ |
|  | *Canis simensis (Csi)* | 1 | 1 | 1 | 1 | - | - |
|  | *Carpocyon* sp. *(Car)* † | 0 | 0 | 1 | 0 | - | - |
|  | *Cerdocyon thous (Cth)* | 5 | 5 | 5 | 5 | 1.02^4, 14^ | - |
|  | *Chrysocyon brachyurus (Cbr)* | 2 | 2 | 2 | 2 | 0.9^15^ | - |
|  | *Cuon alpinus (Cal)* | 4 | 4 | 4 | 4 | - | - |
|  | *Epicyon haydeni (Eha)* † | 0 | 2 | 4 | 1 | - | - |
|  | *Epicyon saevus (Esa)* † | 0 | 1 | 3 | 2 | - | - |
|  | *Lycaon pictus (Lpi)* | 2 | 2 | 2 | 2 | 0.99^4^ | 1.82^43^ |
|  | *Nyctereutes procyonoides (Npr)* | 2 | 2 | 2 | 2 | 0.83^4, 16^ | - |
|  | *Otocyon megalotis (Ome)* | 2 | 2 | 2 | 2 | 1.08^4^ | - |
|  | *Paratomarctus euthos (Peu)* † | 0 | 1 | 0 | 0 | - | - |
|  | *Paratomarctus temerarius (Pte)* † | 0 | 1 | 1 | 1 | - | - |
|  | *Speothos venaticus (Sve)* | 2 | 2 | 2 | 2 | - | - |
|  | *Tomarctus* sp. *(Tom)* † | 0 | 10 | 13 | 7 | - | - |
|  | *Urocyon cinereoargenteus (Uci)* | 4 | 4 | 4 | 4 | -0.26^4^ | 1.62^43^ |
|  | *Vulpes lagopus (Vla)* | 2 | 2 | 2 | 2 | - | - |
|  | *Vulpes velox (Vve)* | 2 | 2 | 2 | 2 | 1.27^4^ | 1.78^44^ |
|  | *Vulpes vulpes (Vvu)* | 3 | 3 | 3 | 3 | 0.71^4, 17, 18^ | 1.68^43^ |
| **Creodonta** | *Patriofelis sp. (Pat)* † | 0 | 0 | 1 | 0 | - | - |
| **Felidae** | *Acinonyx jubatus (Aju)* | 5 | 5 | 5 | 5 | 0.59^5, 19, 20^ | 2.02^43^ |
|  | *Homotherium* sp. *(Hom)* † | 0 | 2 | 1 | 0 | - | - |
|  | *Leptailurus serval (Lse)* | 2 | 2 | 2 | 2 | - | - |
|  | *Lynx rufus (Lru)* | 4 | 4 | 4 | 4 | 0.58^4, 21^ | - |
|  | *Machairodus* sp. *(Mac)* † | 1 | 1 | 4 | 4 | - | - |
|  | *Megantereon* sp. *(Meg)* † | 0 | 2 | 3 | 2 | - | - |
|  | *Neofelis nebulosa (Nne)* | 1 | 1 | 1 | 1 | 0.29^22^ | - |
|  | *Panthera leo (Ple)* | 5 | 5 | 5 | 5 | 0.72^4^ | 1.74^43^ |
|  | *Panthera onca (Pon)* | 4 | 4 | 4 | 4 | 0.51^4, 23^ | - |
|  | *Panthera pardus (Ppa)* | 6 | 6 | 6 | 6 | 1.02^4, 24^ | 1.78^43^ |
|  | *Panthera tigris (Pti)* | 4 | 4 | 4 | 4 | 1.21^25, 26^ | 1.75^43^ |
|  | *Promegantereon ogygia (Pro)* † | 1 | 3 | 5 | 2 | - | - |
|  | *Pseudaelurus* sp. *(Pse)* † | 0 | 3 | 3 | 0 | - | - |
|  | *Puma concolor (Pco)* | 4 | 4 | 4 | 4 | 0.46^27, 28^ | - |
|  | *Smilodon* sp. *(Smi)* † | 0 | 4 | 5 | 3 | - | - |
|  | *Uncia uncia (Uun)* | 4 | 4 | 4 | 4 | 0.5^29, 30^ | - |
| **Hyaenidae** | *Crocuta crocuta (Ccr)* | 5 | 5 | 5 | 5 | 1.05^5, 31^ | 1.78^43^ |
|  | *Hyaena brunnea (Hbr)* | 1 | 1 | 1 | 1 | 1.46^32^ | - |
|  | *Hyaena hyaena (Hhy)* | 2 | 2 | 2 | 2 | 1.28^4^ | 1.7^43^ |
|  | *Pachycrocuta brevirostris (Pbr)* † | 0 | 0 | 1 | 0 | - | - |
|  | *Pliocrocuta perrieri (Ppe)* † | 0 | 0 | 1 | 0 | - | - |
| **Mustelidae** | *Eira barbara (Eba)* | 2 | 2 | 2 | 2 | 0.84^33^ | - |
|  | *Lontra canadensis (Lca)* | 2 | 2 | 2 | 2 | - | - |
|  | *Meles meles (Mme)* | 1 | 1 | 1 | 1 | 0.68^4, 34, 35^ | 1.48^45^ |
| **Nimravidae** | *Dinictis* sp. *(Din)* † | 2 | 3 | 3 | 1 | - | - |
|  | *Hoplophoneus* sp. *(Hop)* † | 0 | 0 | 3 | 2 | - | - |
|  | *Nimravus* sp. *(Nim)* † | 0 | 1 | 0 | 0 | - | - |
|  | *Pogonodon* sp. *(Pog)* † | 0 | 0 | 1 | 0 | - | - |
| **Procyonidae** | *Bassariscus astutus (Bas)* | 1 | 1 | 1 | 1 | - | - |
|  | *Nasua nasua (Nna)* | 1 | 1 | 1 | 1 | - | 1.43^43^ |
|  | *Potos flavus (Pfl)* | 3 | 3 | 3 | 3 | 0.31^36^ | - |
|  | *Procyon lotor (Plo)* | 3 | 3 | 3 | 3 | 0.39^37^ | 1.04^43^ |
| **Ursidae** | *Ailuropoda melanoleuca (Ame)* | 3 | 4 | 4 | 4 | -0.22^1^ | - |
|  | *Arctodus simus (Asi)* † | 0 | 0 | 2 | 2 | - | - |
|  | *Cephalogale* sp. *(Cep)* † | 0 | 0 | 0 | 1 | - | - |
|  | *Helarctos malayanus (Hma)* | 1 | 1 | 1 | 1 | 0.16^38^ | - |
|  | *Hemicyon* sp. *(Hem)* † | 0 | 1 | 2 | 0 | - | - |
|  | *Indarctos* sp. *(Ind)* † | 0 | 0 | 0 | 1 | - | - |
|  | *Melursus ursinus (Mur)* | 3 | 3 | 3 | 3 | - | - |
|  | *Tremarctos ornatus (Tor)* | 1 | 1 | 1 | 1 | - | - |
|  | *Ursus americanus (Uam)* | 3 | 3 | 3 | 3 | 0.12^39^ | 1.68^43^ |
|  | *Ursus arctos (Uar)* | 4 | 4 | 4 | 4 | 0.61^40, 41, 42^ | 1.68^43^ |
|  | *Ursus maritimus (Uma)* | 4 | 4 | 4 | 4 | - | 1.7^43^ |
|  | *Ursus spelaeus (Usp)* † | 0 | 1 | 6 | 5 | - | - |
|  | *Ursus thibetanus (Uth)* | 3 | 3 | 3 | 3 | - | - |

REFERENCES

1. Johnson KG, Schaller, GB, Hu J (1988) Comparative behavior of red and giant pandas in the Wolong reserve, China. J Mamm 69: 552–564.

2. Zhang Z, Hu J, Yang J, Li M, Wei F (2009) Food habits and space-use of red pandas *Ailurus fulgens* in the Fengtongzhai Nature Reserve, China: food effects and behavioural responses. Acta Theriol 54: 225–234.

3. Wei F, Zhang Z (2011) Red Panda Ecology. In: Glastston AR, editor. Red Panda: biology and conservation of the first panda. London, UK: Academic Press. 488 p.

4. Harris MA, Steudel K (1997) Ecological correlates of hind-limb length in the Carnivora. J Zool 241: 381–408.

5. Garland TJ (1983) Scaling the ecological cost of transport to body mass in terrestrial mammals. Am Nat 121: 571–587.

6. Atwood TC, Weeks HP, Gehring TM (2004) Spatial ecology of coyotes along a suburban-to-rural gradient. J Wildl Manage 68: 1000–1009.

7. Andelt WF, Gipson PS (1979) Home Range, Activity, and Daily Movements of Coyotes. J Wildl Manage 43: 944–951.

8. Springer JT (1982) Movement Patterns of Coyotes in South Central Washington. J Wildl Manage 46: 191–200.

9. Jedrzejewski W, Schmidt K, Theuerkauf J, Jedrzejewska B, Okarma H (2001) Daily movements and territory use by radio-collared wolves (*Canis lupus*) in Bialowieza Primeval Forest in Poland. Can J Zool 79: 1993–2004.

10. Kusak J, Skrbinsek AM, Huber D (2005) Home ranges, movements, and activity of wolves (*Canis lupus*) in the Dalmatian part of Dinarids, Croatia. Eur J Wildl Res 51: 254–262.

11. Ciucci P, Boitani L, Francisci F, Andreoli G (1997) Homer range, activity and movements of a wolf pack in central Italy. J Zool 243: 803–819.

12. Ferguson JWH, Nel JAJ, de Wet MJ (1983) Social organization and movement patterns of black-backed jackals *Canis mesomelas* in South Africa. J Zool 199: 487–502.

13. Ferguson JWH, Galpin JS, de Wet MJ (1988) Factors affecting the activity patterns of black-backed jackals *Canis mesomelas*. J Zool 214: 55–69.

14. Juarez KM, Marinho-Filho J (2002) Diet, habitat use, and home range of sympatric canids in central Brazil. J Mammal 83: 925–933.

15. Bandeira de Melo LF, Lima Sábato MA, Vaz Magni EM, Young RJ, Coelho CM (2007) Secret lives of maned wolves (*Chrysocyon brachyurus* Illiger 1815): as revealed by GPS tracking collars. J Zool 271: 27–36.

16. Drygala F, Zoller H, Stier N, Mix H, Roth M (2008) Ranging and parental care of the raccoon dog *Nyctereutes procyonoides* during pup rearing. Acta Theriol 53: 111–119.

17. Woollard T, Harris S (1990) A Behavioural Comparison of Dispersing and Non-Dispersing Foxes (*Vulpes vulpes*) and an Evaluation of Some Dispersal Hypotheses. J Anim Ecol 59: 709–722.

18. Adkins CA, Stott P (1998) Home ranges, movements and habitat associations of red foxes *Vulpes vulpes* in suburban Toronto, Ontario, Canada. J Zool 244: 335–346.

19. Marker LL (2002) Aspects of Cheetah (*Acinonyx jubatus*) Biology, Ecology and Conservation Strategies on Namibian Farmlands. PhD Thesis. Lady Margaret Hall, University of Oxford Trinity Term.

20. Houser A, Somers MJ, Boast LK (2009) Home range use of free-ranging cheetah on farm and conservation land in Botswana. South Afr J Wildl Res 39: 11–22.

21. Lawhead DN (1984) Bobcat *Lynx rufus* home range, density and habitat preference in south-central Arizona. Southwest Nat 29: 105–113.

22. Grassman LIJ, Tewes ME, Silvy NJ, Kreetiyutanont K (2005) Ecology of three sympatric felids in a mixed evergreen forest in north-central Thailand. J Mammal 86: 29–38.

23. Schaller GB, Crawshaw PGJ (1980) Movement patterns of jaguar. Biotropica 12: 161–168.

24. Odden M, Wegge P (2005) Spacing and activity patterns of leopards *Panthera pardus* in the Royal Bardia National Park, Nepal. Wildl Biol 11: 145–152.

25. Sunqist ME (1981) The Social Organization of Tigers (*Panthera tigris*) in Royal Chitawan National Park, Nepal. Smithson Contrib Zool 336: 98 p.

26. Carroll C, Miquelle DG (2006) Spatial viability analysis of Amur tiger *Panthera tigris altaica* in the Russian Far East: the role of protected areas and landscape matrix in population persistence. J Appl Ecol 43: 1056–1068.

27. Hemker TP, Lindzey FG, Ackerman BB (1984) Population Characteristics and Movement Patterns of Cougars in Southern Utah. J Wildl Manage 48: 1275–1284.

28. Beier P, Choate D, Barrett RH (1995) Movement Patterns of Mountain Lions during Different Behaviors. J Mammal 76: 1056–1070.

29. Jackson R, Ahlborn G (1989) Snow Leopards (*Panthera uncia*) in Nepal - Home Range and Movements. Natl Geogr Res 5: 161–175.

30. McCarthy TM, Fuller TK, Munkhtsog B (2005) Movements and activities of snow leopards in Southwestern Mongolia. Biol Conserv 124: 527–537.

31. Kolowski JM, Katan D, Theis KR, Holekamp KE (2007) Daily patterns of activity in the spotted hyena. J Mammal 88: 1017–1028.

32. Maude G (2005) The comparative ecology of the brown hyaena (*Hyaena brunnea*) in Makgadikgadi National Park and neighbouring community cattle area in Bostwana. Pretoria: University of Pretoria eds. 338 p.

33. Konecny MJ (1989) Movement patterns and food habits of four sympatric carnivore species in Belize, Central America. In:  [Redford](http://www.google.es/search?hl=es&tbo=p&tbm=bks&q=inauthor:"Kent+Hubbard+Redford") KH, [Eisenberg](http://www.google.es/search?hl=es&tbo=p&tbm=bks&q=inauthor:"John+Frederick+Eisenberg") JF, editors. Advances in Neotropical Mammalogy. Gainesville, Fla, USA: Sandhill Crane Press. pp. 243–264.

34. Rosalino LM, Macdonald DW, Santos-Reis M (2005) Activity rhythms, movements and patterns of sett use by badgers, *Meles meles*, in a Mediterranean woodland. Mammalia 69: 395–408.

35. Kowalczyk R, Zalewski A, Jedrzejewska B (2006) Daily movement and territory use by badgers *Meles meles* in Bialowieza Primeval Forest, Poland. Wildl Biol 12: 285–391.

36. Julien-Laferriere D (1993) Radio-tracking observations on ranging and foraging patterns by kinkajous (*Potos flavus*) in French Guiana. J Trop Ecol 9: 19–32.

37. Greenwood RJ (1982) Nocturnal Activity and Foraging of Prairie Raccoons (*Procyon lotor*) in North Dakota. Am Midl Nat 107: 238–243.

38. Wong ST, Servheen CW, Ambu L (2004) Home range, movement and activity patterns, and bedding sites of Malayan sun bears *Helarctos malayanus* in the Rainforest of Borneo. Biol Conserv 119: 169–181.

39. Amstrup SC, Beecham J (1976) Activity Patterns of Radio-Collared Black Bears in Idaho. J Wildl Manage 40: 340–348.

40. Craighead FCJ (1976) Grizzly Bear Ranges and Movement as Determined by Radiotracking. In: Bears: Their Biology and Management, Vol. 3, A Selection of Papers from the Third International Conference on Bear Research and Management, Binghamton, New York, USA, and Moscow, U.S.S.R., June 1974. IUCN Publications New Series no. 40. pp. 97–109.

41. Blanchard BM, Knight RR (1991) Movements of Yellowstone Grizzly Bears. Biol Conserv 58: 41–67.

42. Huber D, Roth HU (1993) Movements of European brown bears in Croatia. Acta Theriol 38: 151-159.

43. Christiansen P (2002) Locomotion in terrestrial mammals: the influence of body mass, limb length and bone proportions on speed. Zool J Linn Soc 136: 685–714.

44. Sillero-Zubiri C, Hoffmann M, Macdonald DW (2004). Canids: Foxes, Wolves, Jackals and Dogs. Status Survey and Conservation Action Plan. IUCN/SSC Canid Specialist Group. Gland, Switzerland and Cambridge, UK. 430 p.

45. Garland TJ, Janis CM (1993) Does metatarsal/femur ratio predict maximal running speed in cursorial mammals? J Zool 229: 133–151.

TABLE S2.

| **Species** | **ID** | **Host Institution** |
| --- | --- | --- |
| *Acinonyx jubatus* | 119654 | AMNH |
| *Acinonyx jubatus* | 119655 | AMNH |
| *Acinonyx jubatus* | 119656 | AMNH |
| *Acinonyx jubatus* | 1940.1.20.17 | NHM |
| *Acinonyx jubatus* | 1962.7.6.15 | NHM |
| *Ailuropoda melanoleuca* | 147746 | AMNH |
| *Ailuropoda melanoleuca* | 110454 | AMNH |
| *Ailuropoda melanoleuca* | 89028 | AMNH |
| *Ailuropoda melanoleuca** | 89030 | AMNH |
| *Ailurus fulgens* | 185346 | AMNH |
| *Ailurus fulgens* | 80164 | AMNH |
| *Bassariscus astutus* | 182560 | AMNH |
| *Canis adustus* | 114174 | AMNH |
| *Canis aureus* | 187714 | AMNH |
| *Canis aureus* | 54516 | AMNH |
| *Canis latrans* | 123183 | AMNH |
| *Canis latrans* | 1317 | AMNH |
| *Canis latrans* | 1316 | AMNH |
| *Canis latrans* | 141170 | AMNH |
| *Canis latrans* | 141153 | AMNH |
| *Canis lupus* | 98226 | AMNH |
| *Canis lupus* | 98227 | AMNH |
| *Canis lupus* | 98225 | AMNH |
| *Canis lupus* | 134941 | AMNH |
| *Canis lupus* | 134942 | AMNH |
| *Canis mesomelas* | 34734 | AMNH |
| *Canis mesomelas* | 187712 | AMNH |
| *Canis mesomelas* | 187713 | AMNH |
| *Canis mesomelas* | 114228 | AMNH |
| *Canis simensis* | 81001 | AMNH |
| *Cerdocyon thous* | 134049 | AMNH |
| *Cerdocyon thous* | 214709 | AMNH |
| *Cerdocyon thous* | 214703 | AMNH |
| *Cerdocyon thous* | 209123 | AMNH |
| *Cerdocyon thous* | 209128 | AMNH |
| *Chrysocyon brachyurus* | 133941 | AMNH |
| *Chrysocyon brachyurus* | 133940 | AMNH |
| *Crocuta crocuta* | 35358 | AMNH |
| *Crocuta crocuta* | 83593 | AMNH |
| *Crocuta crocuta* | 52097 | AMNH |
| *Crocuta crocuta* | 187769 | AMNH |
| *Crocuta crocuta* | 187776 | AMNH |
| *Cuon alpinus* | 102083 | AMNH |
| *Cuon alpinus* | 54976 | AMNH |
| *Cuon alpinus* | 54984 | AMNH |
| *Cuon alpinus* | 54842 | AMNH |
| *Eira barbara* | 214736 | AMNH |
| *Eira barbara* | 23487 | AMNH |
| *Helarctos malayanus* | 35364 | AMNH |
| *Hyaena brunnea* | 1962-7.23.1 | NHM |
| *Hyaena hyaena* | 244436 | AMNH |
| *Hyaena hyaena* | 54512 | AMNH |
| *Leptailurus serval* | 119207 | AMNH |
| *Leptailurus serval* | 27837 | AMNH |
| *Lutra canadensis* | 165762 | AMNH |
| *Lutra canadensis* | 182561 | AMNH |
| *Lycaon pictus* | 82085 | AMNH |
| *Lycaon pictus* | 85154 | AMNH |
| *Lynx rufus* | 119206 | AMNH |
| *Lynx rufus* | 208417 | AMNH |
| *Lynx rufus* | 128527 | AMNH |
| *Lynx rufus* | 171361 | AMNH |
| *Meles meles* | 70604 | AMNH |
| *Melursus ursinus* | 150205 | AMNH |
| *Melursus ursinus* | 54465 | AMNH |
| *Melursus ursinus* | 54464 | AMNH |
| *Nasua nasua* | 214722 | AMNH |
| *Neofelis nebulosa* | 238650 | AMNH |
| *Nyctereutes procyonoides* | 249766 | AMNH |
| *Nyctereutes procyonoides* | 249767 | AMNH |
| *Otocyon megalotis* | 233011 | AMNH |
| *Otocyon megalotis* | 63993 | AMNH |
| *Panthera leo* | 1952.10.20.13 | NHM |
| *Panthera leo* | 112.a | NHM |
| *Panthera leo* | 1857.2.24.1 | NHM |
| *Panthera leo* | 75.1998 | NHM |
| *Panthera leo* | 75.945 | NHM |
| *Panthera onca* | 35571 | AMNH |
| *Panthera onca* | 139959 | AMNH |
| *Panthera onca* | 135928 | AMNH |
| *Panthera onca* | 1858.5.26.9 | NHM |
| *Panthera pardus* | 209087 | AMNH |
| *Panthera pardus* | 1940.1.20.18 | NHM |
| *Panthera pardus* | 115p | NHM |
| *Panthera pardus* | 1851.2.17.3 | NHM |
| *Panthera pardus* | 1940.1.20.20 | NHM |
| *Panthera pardus* | 1849.6.20.2 | NHM |
| *Panthera tigris* | 113743 | AMNH |
| *Panthera tigris* | 113748 | AMNH |
| *Panthera tigris* | 135846 | AMNH |
| *Panthera tigris* | 85404 | AMNH |
| *Potos flavus* | 266597 | AMNH |
| *Potos flavus* | 265959 | AMNH |
| *Potos flavus* | 266599 | AMNH |
| *Procyon lotor* | 173897 | AMNH |
| *Procyon lotor* | 147436 | AMNH |
| *Procyon lotor* | 237438 | AMNH |
| *Puma concolor* | 1335 | AMNH |
| *Puma concolor* | 90213 | AMNH |
| *Puma concolor* | 14026 | AMNH |
| *Puma concolor* | 135341 | AMNH |
| *Speothos venaticus* | 52.1086 | NHM |
| *Speothos venaticus* | 1966.1.24.1 | NHM |
| *Tremarctos ornatus* | 81.784 | NHM |
| *Uncia uncia* | 207704 | AMNH |
| *Uncia uncia* | 266952 | AMNH |
| *Uncia uncia* | 119662 | AMNH |
| *Uncia uncia* | 100110 | AMNH |
| *Urocyon cinereoargenteus* | 35695 | AMNH |
| *Urocyon cinereoargenteus* | 90134 | AMNH |
| *Urocyon cinereoargenteus* | 148799 | AMNH |
| *Urocyon cinereoargenteus* | 137028 | AMNH |
| *Ursus americanus* | 128521 | AMNH |
| *Ursus americanus* | 98950 | AMNH |
| *Ursus americanus* | 45149 | AMNH |
| *Ursus arctos* | 14054 | AMNH |
| *Ursus arctos* | 135502 | AMNH |
| *Ursus arctos* | 45150 | AMNH |
| *Ursus arctos* | 70254 | AMNH |
| *Ursus maritimus* | 35065 | AMNH |
| *Ursus maritimus* | 31573 | AMNH |
| *Ursus maritimus* | 215283 | AMNH |
| *Ursus maritimus* | 75244 | AMNH |
| *Ursus thibetanus* | 70320 | AMNH |
| *Ursus thibetanus* | 80248 | AMNH |
| *Ursus thibetanus* | 23086 | AMNH |
| *Vulpes lagopus* | 28117 | AMNH |
| *Vulpes lagopus* | 28116 | AMNH |
| *Vulpes velox* | 35392 | AMNH |
| *Vulpes velox* | 100215 | AMNH |
| *Vulpes vulpes* | 69550 | AMNH |
| *Vulpes vulpes* | 128487 | AMNH |
| *Vulpes vulpes* | 128486 | AMNH |

TABLE S3.

| **Species** | **ID** | **Host Institution** |
| --- | --- | --- |
| **Scapula** |  |  |
| *Amphicyon* sp. | Cast | AMNH |
| *Barbourofelis lovei* | 125115 | AMNH |
| *Dinictis* sp. | 38805 R | AMNH |
| *Dinictis* sp. | 38805 L | AMNH |
| *Machairodus aphanistus* | B-6043 | MNCN |
| *Promegantereon ogygia* | BAT-1-'05-E3-41 | MNCN |
| **Humerus** |  |  |
| *Aelurodon ferox* | 27479 | AMNH |
| *Aelurodon taxoides* | 67481 | AMNH |
| *Aelurodon taxoides* | 30902 | AMNH |
| *Aelurodon taxoides* | 67442 | AMNH |
| *Amphicyon* sp. | Cast | AMNH |
| *Amphicyon ingens* | 68118-B | AMNH |
| *Amphicyon* sp. | 617-27298 | AMNH |
| *Amphicyon* sp. | 68100 | AMNH |
| *Amphicyon* sp. | 68108 | AMNH |
| *Amphicyon* sp. | 68124 | AMNH |
| *Barbourofelis lovei* | 125115 | AMNH |
| *Daphoenus* sp. | 11857 | AMNH |
| *Dinictis* sp. | 125652 L | AMNH |
| *Dinictis* sp. | 125652 R | AMNH |
| *Dinictis* sp. | 1396 | AMNH |
| *Epicyon haydeni* | 67403 | AMNH |
| *Epicyon haydeni* | 67603 | AMNH |
| *Epicyon saevus* | 67489 | AMNH |
| *Hemicyon urisnus* | 21101 | AMNH |
| *Homotherium crenatidens* | 7570V | MSN |
| *Ischyrocyon* sp. | 54220-B | AMNH |
| *Ischyrocyon* sp. | 68158-A | AMNH |
| *Machairodus* sp. | M8960 | NHM |
| *Megantereon cultridens* | Se311 L | NMB |
| *Megantereon cultridens* | Se311 R | NMB |
| *Nimravus* sp. | 62151 | AMNH |
| *Paratomarctus euthos* | 67536 | AMNH |
| *Paratomarctus temerarius* | 105340 | AMNH |
| *Promegantereon ogygia* | B-2465 | MNCN |
| *Promegantereon ogygia* | BAT-1-'03-D4-361 | MNCN |
| *Promegantereon ogygia* | BAT-3-'08-70 | MNCN |
| *Pseudaelurus lorteti* | GA5727 | NMB |
| *Pseudaelurus* sp. | 62202 | AMNH |
| *Pseudaelurus transitorius* | GA5728 | NMB |
| *Simocyon batalleri* | B-2390 | MNCN |
| *Simocyon batalleri* | BAT-1'05-C8-22 | MNCN |
| *Smilodon ensenadensis* | 61 | MCNV |
| *Smilodon fatalis* | LB41 | NMB |
| *Smilodon populator* | 54 | SNM |
| *Smilodon populator* | 2 | SNM |
| *Tomarctus* sp. | 67775 | AMNH |
| *Tomarctus* sp. | 67527 | AMNH |
| *Tomarctus* sp. | 67547 | AMNH |
| *Tomarctus* sp. | 67714 | AMNH |
| *Tomarctus* sp. | 67715 | AMNH |
| *Tomarctus* sp. | 67716 | AMNH |
| *Tomarctus* sp. | 67737 | AMNH |
| *Tomarctus* sp. | 67740 | AMNH |
| *Tomarctus* sp. | 67746 | AMNH |
| *Tomarctus* sp. | 67747 | AMNH |
| *Ursus spelaeus* | Jf771 | NMB |
| **Radius** |  |  |
| *Aelurodon ferox* | 27479 L | AMNH |
| *Aelurodon ferox* | 27479 R | AMNH |
| *Aelurodon ferox* | 61746 | AMNH |
| *Aelurodon ferox* | 67459 | AMNH |
| *Aelurodon ferox* | 70624 | AMNH |
| *Aelurodon taxoides* | 67428 | AMNH |
| *Aelurodon taxoides* | 67445 | AMNH |
| *Amphicyon* sp. | Cast | AMNH |
| *Amphicyon ingens* | 68119 | AMNH |
| *Amphicyon ingens* | 68193 | AMNH |
| *Amphicyon major* | 10428 | AMNH |
| *Amphicyon major* | 29617 | AMNH |
| *Amphicyon* sp. | 23391 | AMNH |
| *Amphicyon* sp. | 25422 | AMNH |
| *Amphicyon* sp. | 617-27298 | AMNH |
| *Amphicyon* sp. | 68103-A | AMNH |
| *Amphicyon* sp. | 68109-A | AMNH |
| *Amphicyon* sp. | 68126 | AMNH |
| *Amphicyon* sp. | 68167 | AMNH |
| *Amphicyon* sp. | 68212 | AMNH |
| *Amphicyon* sp. | 68221 | AMNH |
| *Arctodus simus* | 225-2626 | AMNH |
| *Arctodus simus* | 850 | AMNH |
| *Barbourofelis fricki* | 2672 | AMNH |
| *Barbourofelis fricki* | 61998 | AMNH |
| *Barbourofelis fricki* | 61999 | AMNH |
| *Barbourofelis lovei* | 125115 | AMNH |
| *Barbourofelis morrisi* | 61898 | AMNH |
| *Carpocyon tagarctus* | 67565 | AMNH |
| *Dinictis* sp. | 125652 | AMNH |
| *Dinictis* sp. | 62074 | AMNH |
| *Dinictis* sp. | 62147 | AMNH |
| *Epicyon haydeni* | 67404 | AMNH |
| *Epicyon haydeni* | 67406 | AMNH |
| *Epicyon haydeni* | 67407 | AMNH |
| *Epicyon haydeni* | 67607 | AMNH |
| *Epicyon saevus* | 67490 | AMNH |
| *Epicyon saevus* | 67508 | AMNH |
| *Epicyon saevus* | 8305 | AMNH |
| *Hemicyon* sp. | 68176 | AMNH |
| *Hemicyon ursinus* | 21101 | AMNH |
| *Homotherium serum* | 1798V | MSN |
| *Hoplophoneus insolens* | 655 | AMNH |
| *Hoplophoneus primaevus* | 38980 | AMNH |
| *Hoplophoneus* sp. | 62077 | AMNH |
| *Ischyrocyon* sp. | 54220-B | AMNH |
| *Ischyrocyon* sp. | 68152-A | AMNH |
| *Ischyrocyon* sp. | 68217 | AMNH |
| *Machairodus aphanistus* | B-1482 | MNCN |
| *Machairodus aphanistus* | B-2621 | MNCN |
| *Machairodus giganteus* | M8963 | NHM |
| *Machairodus* sp. | 104725 | AMNH |
| *Megantereon cultridens* | Se311 | NMB |
| *Megantereon cultridens* | StV774.1953 | NMB |
| *Megantereon cultridens* | VA1201 | NMB |
| *Borophagus* sp. | 61664 | AMNH |
| *Borophagus* sp. | 67918-A | AMNH |
| *Borophagus* sp. | 67918 | AMNH |
| *Pachycrocuta brevirostris* | 12823 | MSN |
| *Pliocrocuta perrieri* | 107777 | AMNH |
| *Paratomarctus temerarius* | 105347 | AMNH |
| *Patriofelis ferox* | 1507-A | AMNH |
| *Pogonodon* sp. | 1399 | AMNH |
| *Promegantereon ogygia* | B-2207A | MNCN |
| *Promegantereon ogygia* | B-4413 | MNCN |
| *Promegantereon ogygia* | B-4566 | MNCN |
| *Promegantereon ogygia* | BAT-1-'05-D8-30 | MNCN |
| *Promegantereon ogygia* | BAT-3-9-'01 | MNCN |
| *Pseudaelurus* sp. | 61942-A | AMNH |
| *Pseudaelurus* sp. | 62209 | AMNH |
| *Pseudaelurus* sp. | 62225 | AMNH |
| *Simocyon batalleri* | B-3680 | MNCN |
| *Simocyon batalleri* | B-430 | MNCN |
| *Smilodon ensenadensis* | 61 | MCNV |
| *Smilodon fatalis* | LB41 | NMB |
| *Smilodon populator* | 1.7.85-2 | SNM |
| *Smilodon* sp. | MPCB64-624 | MCNV |
| *Smilodon* sp. | SN | AMNH |
| *Tomarctus* sp. | 67749 | AMNH |
| *Tomarctus* sp. | 67751 | AMNH |
| *Tomarctus* sp. | 67752 | AMNH |
| *Tomarctus* sp. | 67754 | AMNH |
| *Tomarctus* sp. | 67756 | AMNH |
| *Tomarctus* sp. | 67757 | AMNH |
| *Tomarctus* sp. | 67758 | AMNH |
| *Tomarctus* sp. | 67528 | AMNH |
| *Tomarctus* sp. | 67529 | AMNH |
| *Tomarctus* sp. | 67548 | AMNH |
| *Tomarctus* sp. | 67550 | AMNH |
| *Tomarctus* sp. | 67552 | AMNH |
| *Tomarctus* sp. | 67717 | AMNH |
| *Ursus spelaeus* | 17484 | NHM |
| *Ursus spelaeus* | 1953 | NMB |
| *Ursus spelaeus* | 1954 | NMB |
| *Ursus spelaeus* | Jf771 | NMB |
| *Ursus spelaeus* | M324 | NHM |
| *Ursus spelaeus* | M462 | NHM |
| **Ulna** |  |  |
| *Aelurodon ferox* | 27479 L | AMNH |
| *Aelurodon ferox* | 27479 R | AMNH |
| *Aelurodon taxoides* | 67943 | AMNH |
| *Aelurodon taxoides* | 67980 | AMNH |
| *Amphicyon* sp. | Cast | AMNH |
| *Amphicyon ingens* | 68117 | AMNH |
| *Amphicyon ingens* | 68169 | AMNH |
| *Amphicyon major* | TD1158 | NMB |
| *Amphicyon* sp. | 68103-H | AMNH |
| *Amphicyon* sp. | 68110-B | AMNH |
| *Amphicyon* sp. | 68110-C | AMNH |
| *Amphicyon* sp. | 68110-D | AMNH |
| *Amphicyon* sp. | 68127 | AMNH |
| *Amphicyon* sp. | 68221 | AMNH |
| *Amphicyon* sp. | 68266 | AMNH |
| *Arctodus simus* | 1077 | AMNH |
| *Arctodus simus* | 2625 | AMNH |
| *Barbourofelis lovei* | 125115 | AMNH |
| *Barbourofelis morrisi* | 61976 | AMNH |
| *Cephalogale* sp. | 54464 | AMNH |
| *Dinictis* sp. | 62147 | AMNH |
| *Epicyon haydeni* | 67611 | AMNH |
| *Epicyon saevus* | 67492 | AMNH |
| *Epicyon saevus* | 8305 | AMNH |
| *Hoplophoneus primaevus* | 38980 | AMNH |
| *Hoplophoneus* sp. | 62077 | AMNH |
| *Indarctos* sp. | 99236 | AMNH |
| *Ischyrocyon* sp. | 54220-B | AMNH |
| *Ischyrocyon* sp. | 68181-B | AMNH |
| *Machairodus aphanistus* | B-2365 | MNCN |
| *Machairodus aphanistus* | B-2624 | MNCN |
| *Machairodus aphanistus* | B-2720 | MNCN |
| *Megantereon cultridens* | Se311 | NMB |
| *Megantereon cultridens* | VA1201 | NMB |
| *Paratomarctus temerarius* | 61071 | AMNH |
| *Promegantereon ogygia* | BAT-1-'02-E7-72 | MNCN |
| *Promegantereon ogygia* | BAT-3-5-38 | MNCN |
| *Smilodon ensenadensis* | 61 | MCNV |
| *Smilodon fatalis* | LB41 | NMB |
| *Smilodon populator* | 2 | SNM |
| *Tomarctus* sp. | 67759 | AMNH |
| *Tomarctus* sp. | 67760 | AMNH |
| *Tomarctus* sp. | 67761 | AMNH |
| *Tomarctus* sp. | 67720 | AMNH |
| *Tomarctus* sp. | 67721 | AMNH |
| *Tomarctus* sp. | 67722 | AMNH |
| *Tomarctus* sp. | 67725-D | AMNH |
| *Ursus spelaeus* | 1953a | NMB |
| *Ursus spelaeus* | 1953b | NMB |
| *Ursus spelaeus* | 1953c | NMB |
| *Ursus spelaeus* | 43752 | NHM |
| *Ursus spelaeus* | Jf771 | NMB |

TABLE S4.

|  | **Scapula**: |
| --- | --- |
| 1 | Most dorsal point of the posterior border. |
| 2 | Ventral boundary of the teres major process. |
| 3 | Distal tip of the acromion process. |
| 4 | Most posterior point of the metacromion. |
| 5 | Point of maximum curvature between the proximal end of the spine and the scapular body. |
| 6 | Point of maximum curvature at the posterior border of the neck. |
| 7 | Most posterior point of the border of the glenoid fossa. |
| 8 | Midpoint of the lateral border of the glenoid fossa. |
| 9 | Midpoint of the medial border of the glenoid fossa. |
| 10 | Most proximal point of the border of the glenoid fossa at the anterior side. |
| 11 | Most anterior point of the glenoid tuberosity. |
| 12 | Point of maximum curvature at the anterior border of the neck. |
|  |  |
|  | **Humerus**: |
| 1 | Most proximal point of the lesser tuberosity. |
| 2 | Most proximal point of the greater tuberosity. |
| 3 | Most anterior point of the greater tuberosity. |
| 4 | Midpoint of the infraspinatus insertion fossa. |
| 5 | Most lateral point of the lateral epicondyle. |
| 6 | Lateral-proximal corner of the capitulum at the anterior side. |
| 7 | Point of maximum curvature of the articular surface at the anterior side of its proximal border. |
| 8 | Medial-proximal corner of the trochlea at the anterior side. |
| 9 | Most medial point of the medial epicondyle. |
| 10 | Most lateral point at the middle of the shaft. |
| 11 | Most anterior point at the middle of the shaft. |
| 12 | Most medial point at the middle of the shaft. |
| 13 | Most distal point of the humeral head. |
| 14 | Medial-proximal corner of the trochlea at the posterior side |
| 15 | Lateral-proximal corner of the capitulum at the posterior side. |
| 16 | Most distal point of the trochlear ridge. |
| 17 | Most posterior point at the middle of the shaft. |
|  |  |
|  | **Radius**: |
| 1 | Most proximal point of the bicipital tuberosity. |
| 2 | Point of maximum curvature of the head border of the radius at the medial side. |
| 3 | Point of maximum curvature of the head border of the radius at the posterior side. |
| 4 | Most proximal point in the radial coronoid process. |
| 5 | Most medial point at the middle of the shaft. |
| 6 | Most posterior point at the middle of the shaft. |
| 7 | Most lateral point at the middle of the shaft. |
| 8 | Most medial point of the distal epiphysis. |
| 9 | Most distal point of the radial styloid process. |
| 10 | Most proximal point of the anterior border of the distal articular surface. |
| 11 | Most lateral point of the anterior border of the distal articular surface. |
| 12 | Most posterior point of the distal epiphysis. |
| 13 | Most lateral point of the distal epiphysis. |
| 14 | Point of maximum curvature of the radius head border at the lateral side. |
| 15 | Most anterior point at the middle of the shaft. |
| 16 | Most proximal point of the radial styloid process at the anterior side. |
|  |  |
|  | **Ulna**: |
| 1 | Most proximal point of the olecranon process. |
| 2 | Most anterior point of the lateral edge of the olecranon process. |
| 3 | Most anterior point of the medial edge of the olecranon process. |
| 4 | Most anterior point of the anconeus process. |
| 5 | Most distal point of the trochlear groove at the lateral side. |
| 6 | Midpoint of the lateral border of the trochelar groove. |
| 7 | Point of maximum curvature between the border of the trochlear groove and the beginning of the lateral coronoid process. |
| 8 | Most lateral point of the lateral coronoid process. |
| 9 | Midpoint of the distal border of the radial groove. |
| 10 | Most anterior point of the medial coronoid process. |
| 11 | Most medial point of the trochlear groove border. |
| 12 | Point of maximum curvature of the medial border of the trochlear groove, where the anconeus process begins. |
| 13 | Most lateral point at the middle of the shaft. |
| 14 | Most anterior point at the middle of the shaft. |
| 15 | Most medial point at the middle of the shaft. |
| 16 | Most anterior point of the distal epiphysis. |
| 17 | Point of maximum curvature at the beginning of the ulnar styloid process. |
| 18 | Most distal point of the ulnar styloid process. |
| 19 | Most posterior point at the middle of the shaft. |
| 20 | Most posterior point of the distal epiphysis. |

TABLE S5.

| **Taxa** | **Stratigraphic range** | **References for phylogenetic position** | **References for stratigraphic range** |
| --- | --- | --- | --- |
| *Aelurodon ferox* | 15 - 12 my | Wang et al. 1999 | Wang et al. 1999 |
| *Aelurodon taxoides* | 12 - 9 my | Wang et al. 1999 | Wang et al. 1999 |
| *Amphicyon* | 23 - 7.2 my | Hunt 1998 | PBDB |
| *Arctodus simus* | MD 5.66 my | Krause et al. 2008 | Krause et al. 2008 |
| *Barbourofelis* | 11 - 6 my | Morlo et al. 2004 | Hunt 1998, Janis et al. 2008 |
| *Borophagus* | 12 - 2 my | Wang et al. 1999 | Wang et al. 1999 |
| *Carpocyon* | 16 - 5 my | Wang et al. 1999 | Wang et al. 1999 |
| *Cephalogale* | 33.9 - 16 my | Mc Lellan and Reiner 1994 | PBDB |
| *Daphoenus* | 39.5 - 27 my | Hunt 1998 | Hunt 1998, Janis et al. 2008 |
| *Dinictis* | 37 - 26 my | Peigné 2003 | Martin 1998, Peigné 2003, Janis et al. 2009 |
| *Epicyon haydeni* | 10 - 5 my | Wang et al. 1999 | Wang et al. 1999 |
| *Epicyon saevus* | 7 - 12 my | Wang et al. 1999 | Wang et al. 1999 |
| *Hemicyon* | 16 - 13.6 my | Mc Lellan and Reiner 1994 | PBDB |
| *Homotherium* | 3 - 0.5 my | Anton et al. 2004 | Turner & Anton 1997 |
| *Hoplophoneus* | 37 - 28 my | Peigné 2003 | Martin 1998, Peigné 2003, Janis et al. 2008 |
| *Indarctos* | 11 - 5.3 my | Mc Lellan and Reiner 1994 | PBDB |
| *Ischyrocyon* | 14 - 8 my | Hunt 1998 | Hunt 1998, Janis et al. 2008 |
| *Machairodus* | 15 - 2 my | Anton et al. 2004 | Turner & Anton 1997 |
| *Megantereon* | 5.3 - 0.78 my | Anton et al. 2004 | PBDB |
| *Nimravus* | 34 - 24 my | Peigné 2003 | Peigné 2003, Martin 1998, Janis et al. 2009 |
| *Pachycrocuta brevirostris* | 3.4 - 0.3 my | Turner et al. 2008 | NOW database |
| *Paratomarctus euthos* | 13 - 9 my | Wang et al. 1999 | Wang et al. 1999 |
| *Paratomarctus temerarius* | 16 - 13 my | Wang et al. 1999 | Wang et al. 1999 |
| *Patriofelis* | 50.3 - 40.4 my | Gunnell 1998 | PBDB |
| *Pliocrocuta perrieri* | 4.2 - 1.2 my | Turner et al. 2008 | NOW database |
| *Pogonodon* | 34 - 23 my | Peigné 2003 | Peigné 2003, Martin 1998, Janis et al. 2008 |
| *Promegantereon ogygia* | 11 - 8.2 my | Salesa et al. 2010 | NOW database |
| *Pseudaelurus* | 20.4 - 4.9 my | Rothwell 2003 | PBDB |
| *Simocyon batalleri* | 11.1 - 8.7 my | Salesa et al. 2011 | Salesa et al. 2011 |
| *Smilodon* | 4.9 - 0.01 my | Anton et al. 2004 | PBDB |
| *Tomarctus* | 16 - 14 my | Wang et al. 1999 | Wang et al. 1999 |
| *Ursus spelaeus* | MD 2.75 my | Krause et al. 2008 | Krause et al. 2008 |
|  |  |  |  |
|  | **Time of divergence** |  | **References for time of divergence** |
| Order Creodonta | 65.1 my | Gunnell 1998 | Nyakatura and Bininda-Emonds 2012 |
| Family Amphicyonidae | 61.5 my | Finarelli & Flynn 2006 | Nyakatura and Bininda-Emonds 2012 |
| Family Barbourofelidae | 20 my | Morlo et al. 2004 | PBDB |
| Family Nimravidae | 37 my | Peigné 2003 | Martin 1998, Peigné 2003, Janis et al. 2008 |
| Subfamily Borophaginae | 34 my | Wang et al. 1999 | Wang et al. 1999 |
| Subfamily Machairondontinae | 15 my | Anton et al. 2004 | Turner & Anton 1997 |
| Subfamily Simocyoninae | 17 my | Salesa et al. 2011 | Salesa et al. 2011, Wallace 2011 |
