# Supplementary figures and images for: A Three-Dimensional Analysis of Morphological Evolution and Locomotor Performance of the Carnivoran Forelimb
Source: PLoS One. 2014 Jan 15;9(1):e85574. doi: 10.1371/journal.pone.0085574 (PMC3893248; doi:10.1371/journal.pone.0085574)

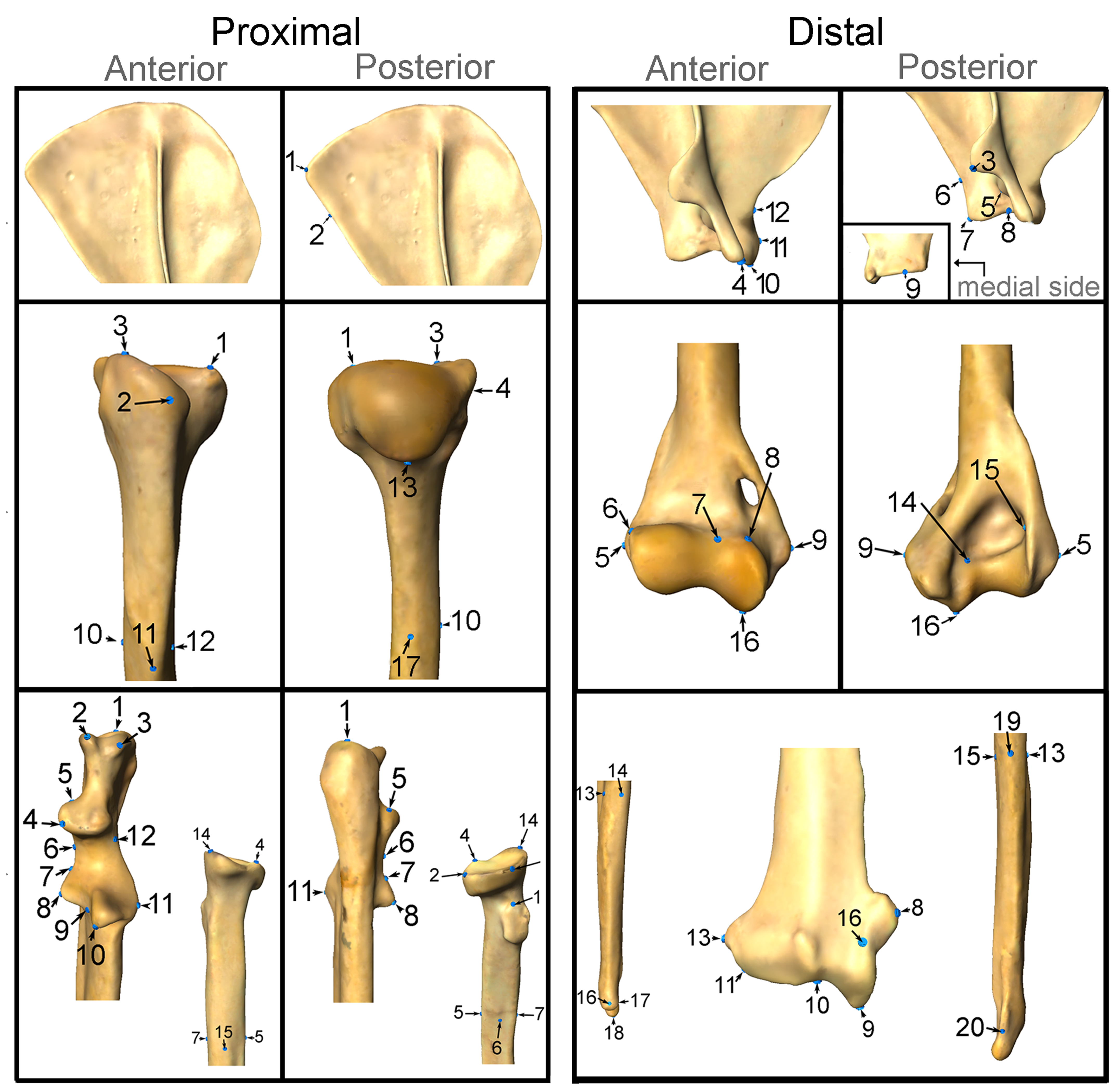

Supplement: Figure S1 — Detailed figure of the landmarks used in this paper. (TIF) [file pone.0085574.s002.tif]

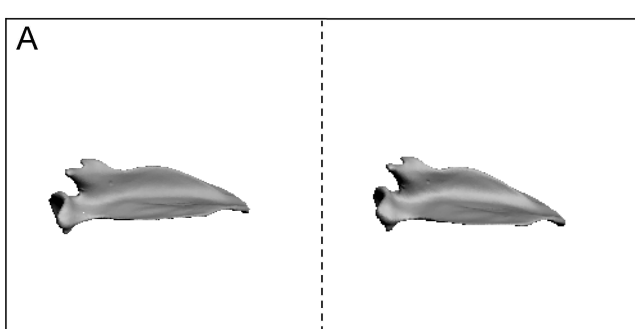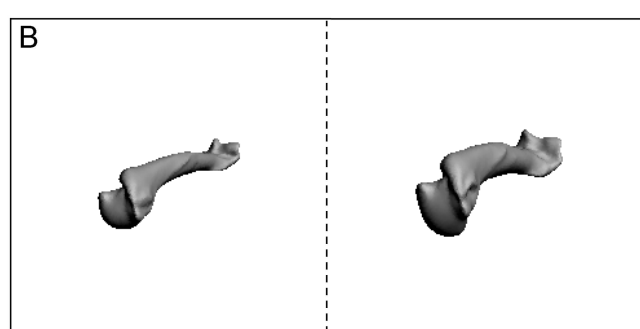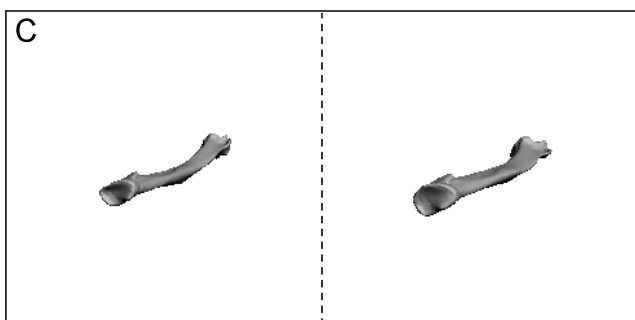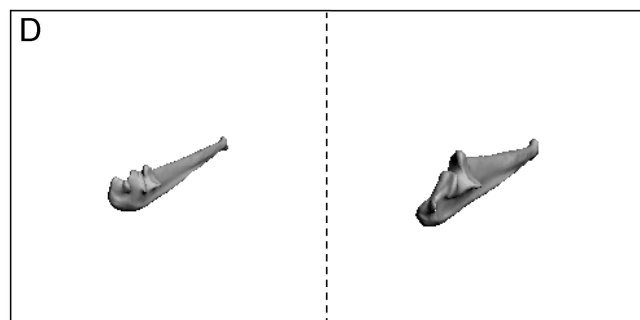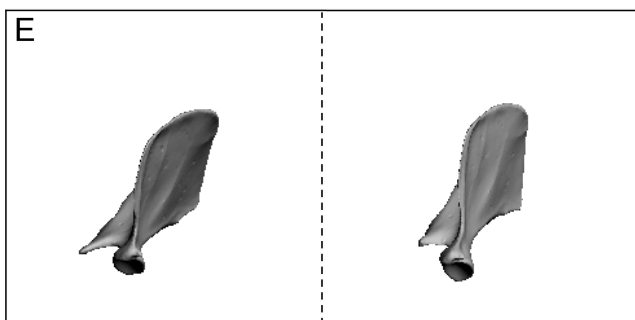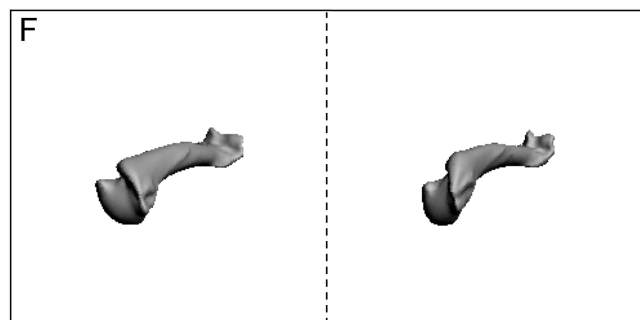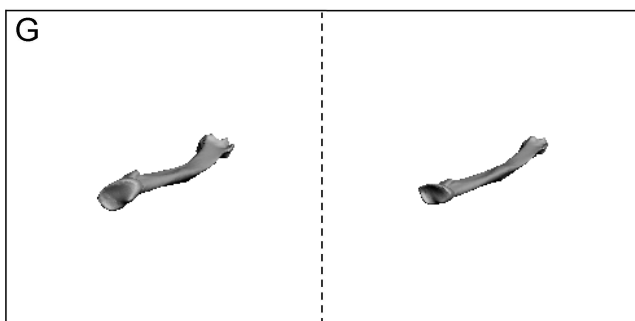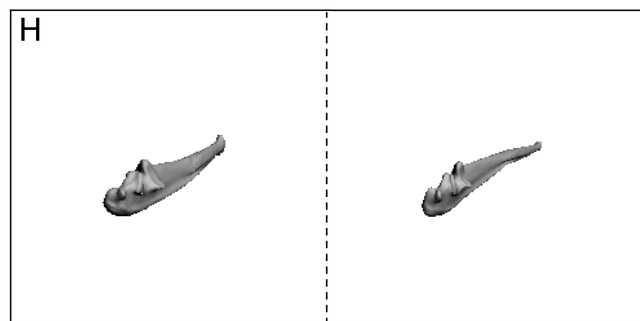

Supplement: File S3 — Interactive three-dimensional models of shape variation in the carnivoran forelimb. Size-related shape changes for scapula (A), humerus (B), radius (C) and ulna (D). Shape changes accounted for by variation in DMD in the scapula (E), humerus (F), radius (G) and ulna (H). Left indicates negative regression scores and right positive ones. (PDF) [file pone.0085574.s004.pdf]

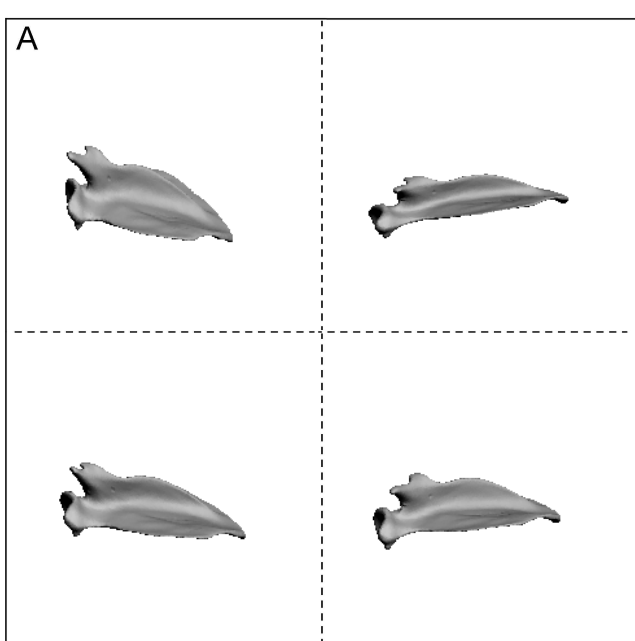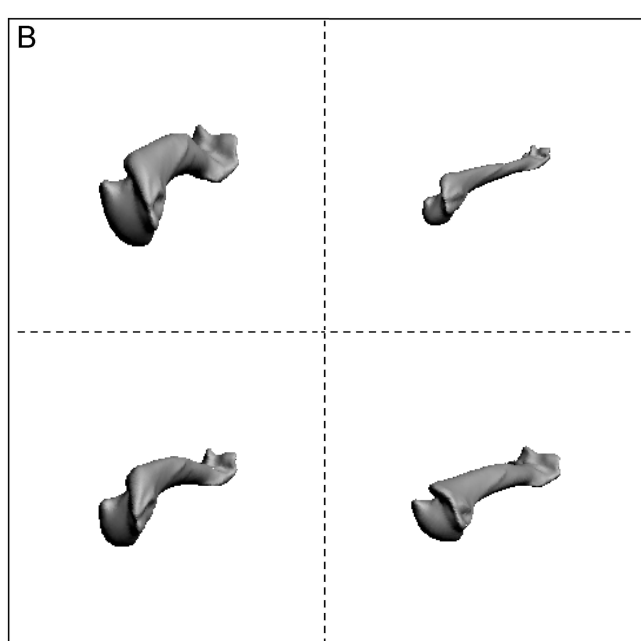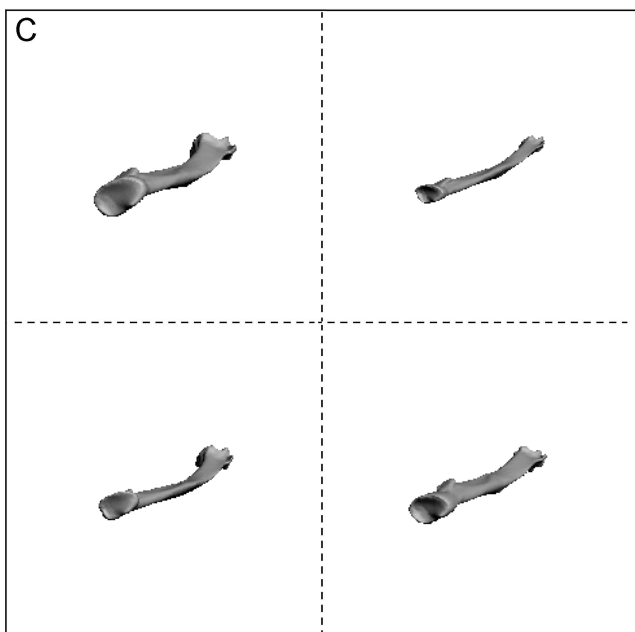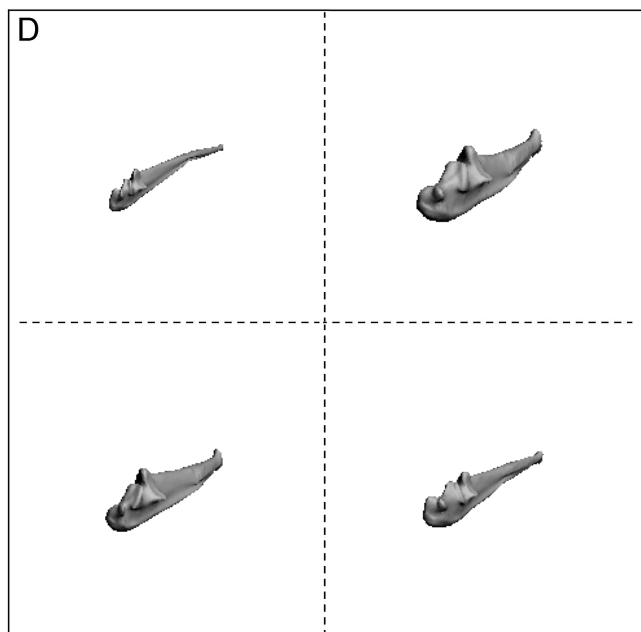

Supplement: File S4 — Three-dimensional models showing the shape changes obtained from the PCA's. A, scapula; B, humerus; C, radius; D, ulna. PC I top, PC II bottom; left for negative scores, right for positive scores. (PDF) [file pone.0085574.s005.pdf]
