# Supplementary material for: A Three-Dimensional Analysis of Morphological Evolution and Locomotor Performance of the Carnivoran Forelimb
Source: PLoS One. 2014 Jan 15;9(1):e85574. doi: 10.1371/journal.pone.0085574 (PMC3893248; doi:10.1371/journal.pone.0085574)
Supplement: File S2 — Nexus file of the composite tree used in this paper. (PDF) [file pone.0085574.s003.pdf]

```
#NEXUS

begin TREES;

	TRANSLATE
		1 Daphoenus,
		2 Amphicyon,
		3 Ischyrocyon,
		4 Tomarctus,
		5 Aelurodon_ferox,
		6 Aelurodon_taxoides,
		7 Paratomarctus_euthos,
		8 Paratomarctus_temerarius,
		9 Carpocyon,
		10 Epicyon_haydeni,
		11 Epicyon_saevus,
		12 Borophagus,
		13 Nasua_nasua,
		14 Bassariscus_astutus,
		15 Potos_flavus,
		16 Procyon_lotor,
		17 Meles_meles,
		18 Eira_barbara,
		19 Lutra_canadensis,
		20 Ailurus_fulgens,
		21 Simocyon_batalleri,
		22 Urocyon_cinereoargenteus,
		23 Vulpes_lagopus,
		24 Vulpes_velox,
		25 Vulpes_vulpes,
		26 Nyctereutes_procyonoides,
		27 Otocyon_megalotis,
		28 Cerdocyon_thous,
		29 Chrysocyon_brachyurus,
		30 Speothos_venaticus,
		31 Canis_adustus,
		32 Canis_mesomelas,
		33 Lycaon_pictus,
		34 Cuon_alpinus,
		35 Canis_simensis,
		36 Canis_aureus,
		37 Canis_latrans,
		38 Canis_lupus,
		39 Acinonyx_jubatus,
		40 Puma_concolor,
		41 Leptailurus_serval,
		42 Lynx_rufus,
		43 Neofelis_nebulosa,
		44 Uncia_uncia,
		45 Panthera_tigris,
		46 Panthera_onca,
		47 Panthera_pardus,
		48 Panthera_leo,
		49 Hyaena_hyaena,
		50 Hyaena_brunnea,
```

51 Pliocrocuta_perrieri,
52 Pachycrocuta_brevirostris,
53 Crocuta_crocuta,
54 Homotherium,
55 Machairodus,
56 Promegantereon_ogygia,
57 Megantereon,
58 Smilodon,
59 Barbourofelis,
60 Pseudaelurus,
61 Hoplophoneus,
62 Nimravus,
63 Pogonodon,
64 Dinictis,
65 Ursus_arctos,
66 Ursus_maritimus,
67 Ursus_spelaeus,
68 Ursus_americanus,
69 Ursus_thibetanus,
70 Melursus_ursinus,
71 Helarctos_malayanus,
72 Tremarctos_ornatus,
73 Arctodus_simus,
74 Ailuropoda_melanoleuca,
75 Indarctos,
76 Cephalogale,
77 Hemicyon,
78 Patriofelis;

TREE 'Untitled Tree+++' =
(78:24.7,((((49:1.5,50:1.5):2.8,(51:3.0,(52:3.1,53:3.4):0.8):0.1):48.7,((62:13.2,(61:9.1,(63:14.0,64:11.0):0.1):0.1):0.1,(59:14.5,(60:15.5,(((54:14.6,55:13.0):0.1,(56:2.8,(57:4.5,58:5.29):4.7):5.1):1.3,((((39:7.0,40:7.0):4.5,42:11.5):4.1,41:15.6):0.7,(43:9.2,((44:3.5,45:3.5):2.6,(46:3.9,(47:2.9,48:2.9):1.0):2.2):3.1):7.1):0.1):4.0):0.1):16.7):15.8):12.0,((1:12.5,(2:15.8,3:15.0):16.5):22.0,((((20:16.9,21:8.6):18.1,(((19:14.7,18:14.7):2.3,17:17.0):14.7,(15:22.0,(13:12.1,(14:12.0,16:12.0):0.1):9.9):9.7):3.3):13.0,(76:17.9,(77:3.6,(74:17.1,(75:11.7,((72:5.66,73:5.56):9.84,(70:6.4,((71:4.8,(68:3.8,69:3.8):1.0):0.1,(67:3.47,(65:1.0,66:1.0):2.6):1.3):1.5):9.1):1.5):0.1):0.1):16.8):14.0):13.4,(((4:2.0,(5:3.0,6:6.0):1.0):0.2,((7:7.0,8:3.0):0.1,(9:11.0,(12:10.0,(10:7.0,11:5.0):0.1):3.9):0.1):0.1):17.8,(22:16.3,((27:16.0,(26:15.9,(25:5.8,(23:3.0,24:3.0):2.8):10.1):0.1):0.1,((33:6.6,30:6.6):2.7,((28:9.0,29:9.0):0.1,((31:4.2,32:4.2):4.8,(34:4.1,(35:3.9,((36:2.4,37:2.4):0.2,38:2.6):1.3):0.2):4.9):0.1):0.2):6.8):0.2):17.7):27.4):0.1):3.5):0.1):21.2;
end;
